# MicroRNAs in Breast Cancer: Biological Functions and Technologies for Experimental and Therapeutic Applications

**DOI:** 10.3390/cancers18162708

**Published:** 2026-08-21

**Authors:** Marios A. Diamantopoulos, Michaela A. Boti, Evangelos Kanellopoulos, Andreas Scorilas

**Affiliations:** Department of Biochemistry and Molecular Biology, Faculty of Biology, National and Kapodistrian University of Athens, Panepistimiopolis, 15701 Athens, Greece; imdiamantop@biol.uoa.gr (M.A.D.); miboti@biol.uoa.gr (M.A.B.); evkanell@biol.uoa.gr (E.K.)

**Keywords:** breast cancer, miRNAs modulation strategies, miRNA sponges, anti-miRNA oligonucleotides, miRNA mimics, CRISPR-Cas systems, breast cancer treatment, miRNA-based therapeutics, delivery systems, molecular oncology

## Abstract

Breast cancer is a highly heterogeneous malignancy that remains one of the leading causes of cancer-related mortality among women worldwide. Despite significant advances in breast cancer research and therapy, disease heterogeneity, treatment resistance, and metastatic progression remain major obstacles to effective disease management. Among the molecular regulators involved in breast cancer, microRNAs (miRNAs) have been recognized as critical post-transcriptional regulators of gene expression, functioning as either oncogenes or tumor suppressors. By modulating the expression of target RNAs, miRNAs control key biological processes involved in tumor initiation and progression, including cell proliferation, apoptosis, angiogenesis, epithelial–mesenchymal transition (EMT), invasion, and metastasis. To investigate miRNA function and explore their therapeutic potential, a wide range of approaches have been developed to modulate miRNA expression. These include gain-of-function strategies, like miRNA mimics, miRNA expression vectors, and CRISPR activation (CRISPRa), as well as loss-of-function approaches, including anti-miRNA oligonucleotides (AMOs), miRNA sponges, CRISPR-Cas9-mediated gene knockout, and CRISPR interference (CRISPRi). This review provides a comprehensive overview of the biological roles of miRNAs in breast cancer and discusses current technologies for miRNA modulation, their molecular mechanisms, experimental and therapeutic applications, and associated limitations. In addition, it summarizes recent advances in miRNA delivery systems, including viral vectors, organic nanoparticles, and inorganic nanocarriers, highlighting their potential to improve delivery efficiency, target specificity, and facilitate clinical translation. Finally, the review discusses future perspectives, emphasizing the transition from single-target interventions toward network-level regulation and the integration of miRNA-based strategies into precision oncology to support the development of more effective breast cancer therapies.

## Simple Summary

Breast cancer is the most common malignancy among women and remains a major cause of cancer-related deaths worldwide. MicroRNAs (miRNAs) are critical regulators of gene expression that play essential roles in maintaining normal cellular function, and their dysregulation has been associated with breast cancer initiation, progression, and therapeutic resistance. Consequently, a variety of approaches have been developed to modulate miRNA expression by either restoring or inhibiting specific miRNAs, enabling researchers to investigate their biological functions and also explore their therapeutic potential. This review summarizes the diverse roles of miRNAs in breast cancer, describes current strategies for miRNA modulation, and discusses recent advances in delivery systems that can support the clinical translation of miRNA-based therapeutics. A better understanding of these technologies may facilitate the development of more precise and effective treatments for breast cancer.

## 1. Introduction

Although breast cancer is among the most extensively studied malignancies, its management remains challenging due to its high molecular heterogeneity, frequent therapeutic resistance, and, in many cases, late-stage diagnosis [1,2]. Over the past decades, advances in molecular oncology have significantly improved our understanding of the genetic and epigenetic alterations that drive breast cancer progression, leading to the identification of key regulatory molecules involved in tumor development [3]. Among these, microRNAs (miRNAs) have been reported as critical regulators of tumorigenesis, underscoring their biological relevance in cancer and the need for further investigation.

These small non-coding RNAs act at the post-transcriptional level by controlling the expression of multiple target RNAs [4,5]. Through this regulatory capacity, miRNAs influence multiple cancer-associated processes, including proliferation, apoptosis, angiogenesis, invasion, metastasis, and resistance to therapy [6]. Importantly, aberrant miRNA expression is a common feature of breast cancer and frequently occurs in a subtype-specific manner, contributing not only to tumor initiation and progression but also to the molecular and phenotypic heterogeneity of the disease [7].

The recognition of miRNAs as central regulators of oncogenic and tumor-suppressive pathways has positioned them as valuable diagnostic and prognostic biomarkers, as well as promising therapeutic targets [8,9,10,11,12]. Unlike traditional targeted therapies that typically inhibit individual molecular targets [13], miRNA-based strategies offer the unique advantage of simultaneously modulating entire regulatory networks. This can be achieved either by restoring tumor-suppressive miRNAs that are lost or downregulated in cancer, or by inhibiting oncogenic miRNAs, thereby partially re-establishing normal cellular functions [14]. As a result, technologies enabling precise miRNA modulation have become essential tools for studying miRNA function, while also providing promising platforms for therapeutic intervention.

In parallel, considerable progress has been made in developing approaches that enable targeted modulation of miRNA expression and activity [15]. Current technologies include synthetic miRNA mimics, anti-miRNA oligonucleotides, miRNA sponges, and more recently, the CRISPR-Cas systems, which allow either transient or stable gain- and loss-of-function modulation of specific miRNAs [15,16]. Furthermore, advances in delivery platforms have addressed several limitations associated with miRNA-based therapeutics, particularly regarding molecular stability, target specificity, biosafety, and efficient in vivo delivery. Together, these developments have expanded the experimental toolkit available for studying miRNA biology and have further highlighted the therapeutic potential of miRNA-based strategies.

This review provides a comprehensive overview of the biological functions of miRNAs in breast cancer, with a particular focus on their roles in key cancer-related processes and their contribution to tumor initiation and progression. In addition, it examines current strategies for miRNA modulation, discusses the therapeutic potential of miRNA-based approaches, and highlights recent advances in delivery technologies. Finally, the review addresses future perspectives, emphasizing emerging technologies that may enable more precise and effective miRNA-based interventions within the framework of precision oncology.

## 2. Epidemiology and Molecular Landscape of Breast Cancer

Breast cancer is the most commonly diagnosed malignancy and one of the leading causes of cancer-related mortality among women worldwide. It is characterized by extensive molecular heterogeneity, which results in substantial variability in tumor behavior, clinical outcomes, and therapeutic responses, complicating disease management [17,18]. Over the past decade, considerable research efforts have been devoted to elucidating the molecular mechanisms underlying breast cancer and to identifying reliable molecular signatures that can improve disease classification, refine prognostic assessment, and guide the development of more effective, personalized therapies.

### 2.1. Epidemiology of Breast Cancer

According to GLOBOCAN 2022 data, breast cancer is the second most commonly diagnosed malignancy worldwide across both sexes, with approximately 2.3 million new cases accounting for 11.6% of all cancer diagnoses in 2022. It is also the fourth leading cause of cancer-related mortality globally [19]. According to the World Health Organization, breast cancer was responsible for approximately 670,000 deaths in 2022 and represented the most frequently diagnosed cancer among women in 157 of 185 countries, accounting for 23.8% of all newly diagnosed cancers and 15.4% of cancer-related deaths among women [20,21].

Current projections suggest that, if present trends persist, the global burden of breast cancer will increase substantially over the coming decades. By 2040, the annual number of newly diagnosed cases is expected to exceed 3 million, representing an increase of more than 40%, while breast cancer-related mortality is projected to rise by over 50%, approaching 1 million deaths annually [22].

### 2.2. Molecular Heterogeneity of Breast Tumors

Breast cancer was initially classified using the TNM staging system, which stratifies tumors according to tumor size (T), lymph node involvement (N), and the presence or absence of distant metastasis (M). Based on these clinicopathological parameters, specific therapeutic strategies, including surgical intervention, chemotherapy, or radiotherapy, were selected [23,24]. Although this system remains essential for clinical decision-making, its inability to capture the molecular diversity of breast tumors highlighted the need for more precise and informative classification systems.

**Figure 1:**
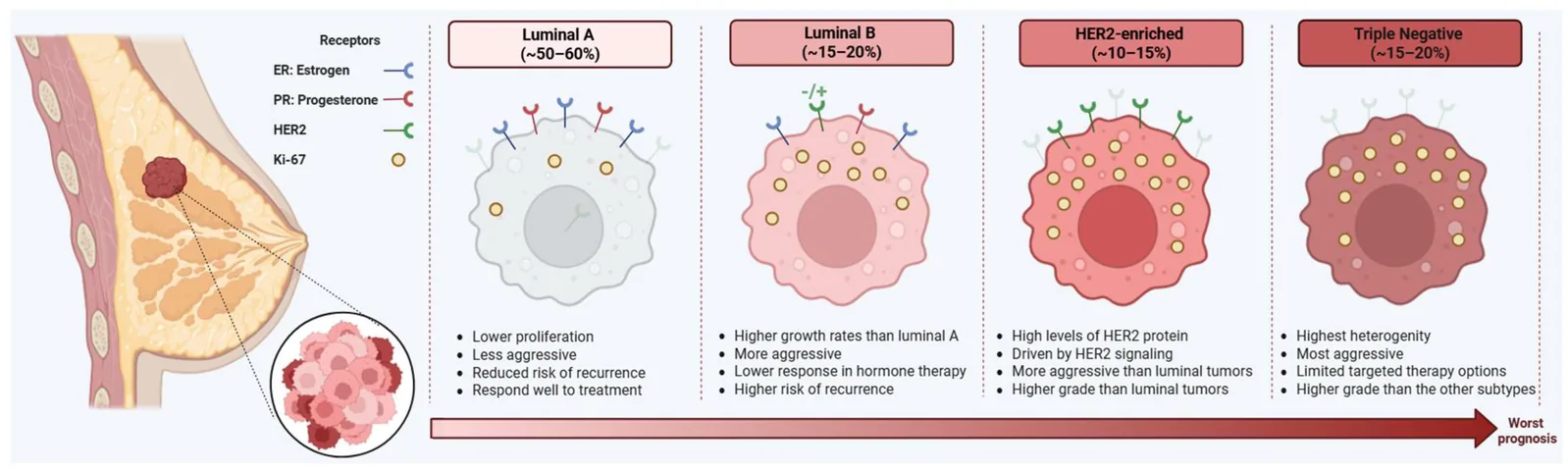
Molecular classification of breast cancer. Breast cancer is classified into four major molecular subtypes based on the expression of estrogen receptor (ER), progesterone receptor (PR) and human epidermal growth factor receptor 2 (HER2). Luminal A tumors are characterized by low proliferative activity and favorable prognosis, whereas luminal B tumors exhibit higher proliferation rates and a less favorable clinical outcome. HER2-positive tumors are driven by HER2 overexpression and are generally associated with more aggressive disease. Triple-negative breast cancer (TNBC), which lacks ER, PR, and HER2 expression, is the most heterogeneous and aggressive subtype, with limited targeted therapeutic options.

Advances in molecular biology and gene expression profiling have significantly improved breast cancer stratification by providing additional molecular information beyond conventional TNM staging. Molecular classification, based primarily on receptor expression status, has enabled a more refined characterization of breast tumors and improved treatment selection. This classification system recognizes four major breast cancer subtypes: luminal A (ER+, PR ≥ 20%, HER2-, Ki-67 < 20%), luminal B (ER+, HER2+/−, PR < 20% or Ki-67 ≥ 20%), HER2-positive (ER−, PR−, HER2+), and triple-negative (ER−, PR−, HER2−) [25,26] (Figure 1). Among these molecular subtypes, the triple-negative (TNBC) subtype is generally characterized by the most aggressive behavior and the poorest prognosis, whereas luminal A tumors typically demonstrate the most favorable prognosis and lowest proliferative activity [27]. Due to the significant heterogeneity TNBC exhibit, these tumors can be further subdivided based on gene expression profiling into six additional subtypes. These TNBC subtypes include basal-like 1 (BL1), basal-like 2 (BL2), immunomodulatory (IM), mesenchymal (M), mesenchymal stem-like (MSL), and luminal androgen receptor (LAR) [28]. Each of these subdivisions exhibits distinct molecular characteristics, phenotypic features, clinical outcomes, and therapeutic sensitivities, underscoring the biological complexity of TNBC.

### 2.3. miRNA Expression Across Breast Cancer Subtypes

miRNAs are key regulators of gene expression that may contribute to the molecular heterogeneity of breast cancer [29]. By regulating their target transcripts, miRNAs control critical cellular processes involved in tumorigenesis, including proliferation, apoptosis, epithelial-to-mesenchymal transition (EMT), invasion, metastasis, and drug resistance [30,31]. Notably, miRNA expression patterns differ among breast cancer subtypes, reflecting their distinct molecular and phenotypic characteristics and, in many cases, contributing to subtype-specific tumor behavior [32,33,34].

Previous studies have identified subtype-specific miRNA expression patterns utilizing breast cancer patient-derived samples, including tissue and blood specimens, as well as established breast cancer cell lines, highlighting the close association between miRNA dysregulation and the distinct biological characteristics of each breast cancer molecular subtype [35]. In luminal breast cancers, miRNA expression patterns are closely linked to hormone receptor signaling pathways. Several miRNAs, including members of the miR-30 family and the miR-99a/let-7c/miR-125b cluster, have been associated with tumor-suppressive functions and the regulation of proliferation and migration. Notably, increased expression of members of the miR-99a/let-7c/miR-125b cluster has been reported in patient-derived luminal A tumor tissues, consistent with the less aggressive phenotype and more favorable prognosis observed in this subtype [36]. In contrast, luminal B tumors exhibit a higher degree of miRNA dysregulation, reflecting their increased proliferative capacity and more aggressive biological behavior. Tissue-based profiling studies further distinguish these subtypes, with patient-derived luminal A tumors exhibiting higher expression of miR-30c-5p and miR-30b-5p, whereas luminal B tumors demonstrate increased expression of miR-182-5p, miR-200b-3p, miR-15b-3p, miR-149-5p, miR-193b-3p, and miR-342-3p/5p (Table 1) [32,37]. Additionally, differential circulating levels of miR-29a, miR-181a, and miR-652 have been reported between luminal A and luminal B subtypes. Notably, these miRNAs are consistently downregulated in both tumor tissues and serum samples from patients with luminal A compared to luminal B tumors [38], further underscoring their potential relevance in subtype discrimination and disease monitoring.

HER2-positive breast cancers also display distinct miRNA expression patterns, which are closely associated with HER2-driven oncogenic signaling [39]. For instance, miR-125b has been reported to be upregulated in HER2-positive tumor tissues [40], while miR-4728-3p, which is encoded within an intronic region of the HER2 gene, appears to be particularly associated with this subtype based on its increased expression in both patient-derived tumor tissues and HER2-positive breast cancer cell lines [41]. Additional dysregulated miRNAs include the upregulated miR-146a-5p, as well as the downregulated miR-181d and miR-195-5p [42]. These miRNAs have been implicated not only in tumor progression but also in therapeutic response, particularly in resistance to HER2-targeted therapies [43]. Nevertheless, despite increasing evidence linking miRNAs to treatment resistance, the precise mechanisms and the causal relationships between specific miRNAs and resistance remain to be fully elucidated, underscoring the need for further functional studies.

TNBC subtype is characterized by a highly dysregulated miRNA landscape. Studies using patient-derived tumor tissues have identified TNBC-associated miRNA expression patterns, including the upregulation of miR-17-5p, miR-20a-5p, miR-92a-3p, and miR-106b-5p, which are not observed in other breast cancer molecular subtypes [44]. In contrast, comparative profiling studies conducted in breast cancer cell lines have identified a distinct set of differentially expressed miRNAs between TNBC and non-TNBC models, including the upregulated miR-138-5p, miR-4324, miR-4800-3p, and miR-6836-3p, as well as the downregulated miR-363-5p, miR-182-5p, miR-141-3p, miR-339-5p, miR-4655-3p, miR-4784, miR-664b-5p, and miR-6787-5p [45]. However, findings derived from cell lines should be interpreted with caution, as these models may not fully recapitulate the molecular heterogeneity, tumor microenvironment, and miRNA expression patterns observed in clinical tumor specimens. Moreover, the substantial biological heterogeneity of TNBC complicates the identification of universally dysregulated miRNAs across all tumors within this subtype.

The heterogeneity of miRNA expression across breast cancer subtypes has important implications for both research and therapeutic development. Due to their ability to regulate multiple targets within oncogenic pathways, miRNAs represent promising candidates for therapeutic intervention. However, the regulatory impact of a given miRNA may vary considerably depending on the molecular subtype [29], highlighting the need for subtype-specific targeting approaches. A comprehensive understanding of subtype-dependent miRNA functions is therefore essential for advancing the mechanistic understanding of breast cancer biology and the development of effective miRNA-based therapeutic approaches.

## 3. Biological Roles of miRNAs in Breast Cancer

miRNAs are involved in a wide range of biological processes essential for cellular development, survival, and function [46]. By recognizing partially complementary sequences within the 3′ untranslated regions (3′UTRs) of target mRNAs, miRNAs promote mRNA degradation or inhibit translation, thereby controlling protein production [47,48,49]. Their ability to post-transcriptionally regulate gene expression underscores their critical roles in the pathogenesis of multiple malignancies, including breast cancer. In this regard, these molecules can function either as oncogenes or tumor suppressors by modulating the expression of key target genes involved in cell proliferation, apoptosis, differentiation, invasion, metastasis, angiogenesis, and EMT [6]. This section summarizes the functional roles of miRNAs in key biological processes associated with tumor initiation, progression, and therapeutic resistance in breast cancer.

### 3.1. Cell Proliferation and Survival

**Figure 2:**
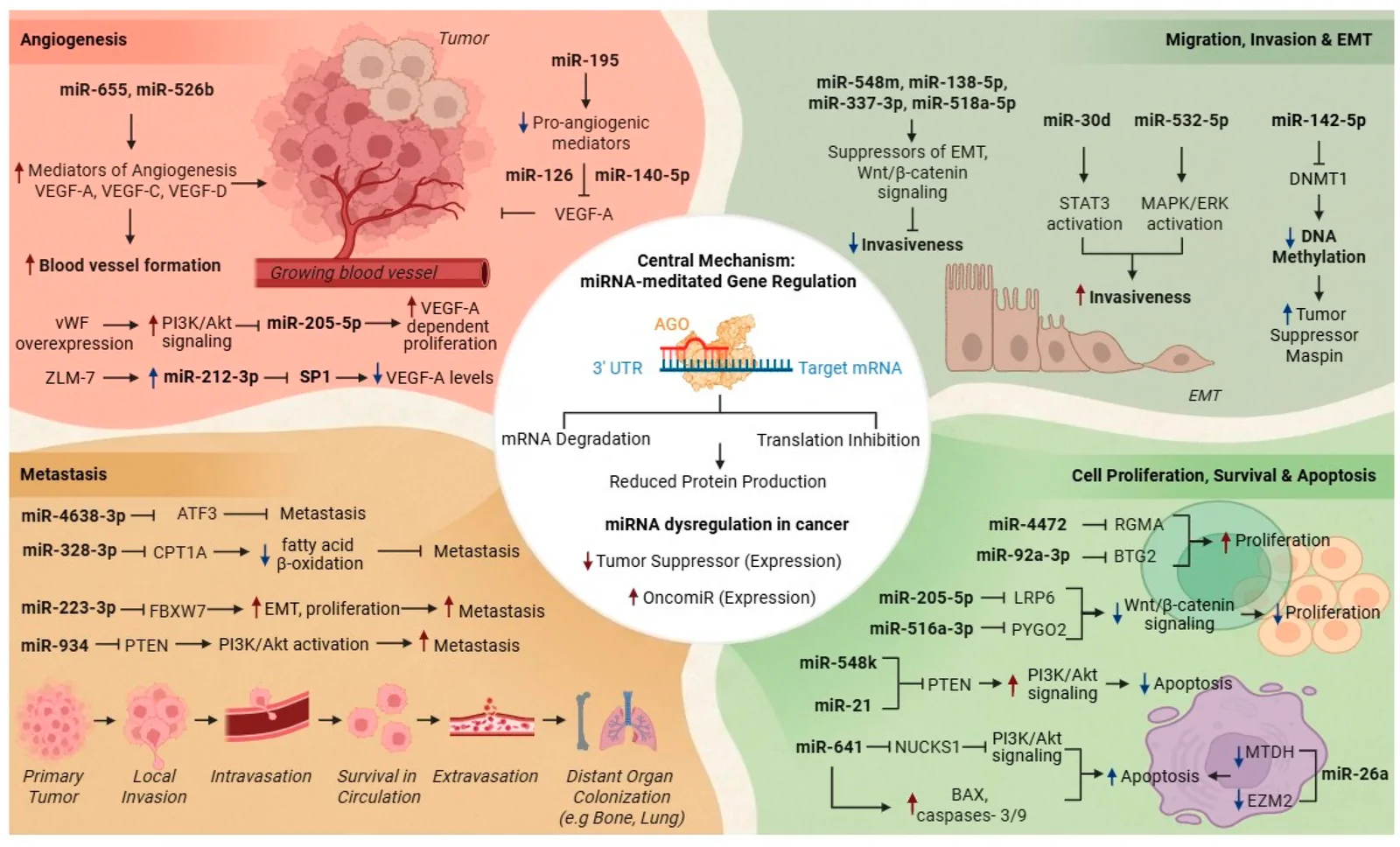
Role of miRNAs in breast cancer. miRNAs regulate gene expression by inducing mRNA degradation or translational repression. Dysregulation of tumor-suppressive miRNAs and oncogenic miRNAs has been associated with angiogenesis, cancer cell proliferation, survival, apoptosis, migration, invasion, epithelial–mesenchymal transition (EMT), and metastasis through modulation of key signaling pathways. Representative miRNAs and their principal molecular targets are shown. Arrows (↑/↓) indicate increased or decreased expression or activity, and blunt-ended lines (⊣) indicate inhibitory interactions. AGO, Argonaute protein.

Sustained proliferation and enhanced cellular survival are hallmarks of cancer, contributing to tumor development and progression [50]. Under physiological conditions, these processes are tightly regulated to maintain tissue homeostasis. However, dysregulation of key regulatory molecules can promote uncontrolled proliferation and resistance to cell death. Today, miRNAs are recognized as critical regulators of these processes through their ability to modulate genes involved in cell cycle progression, oncogenic signaling pathways, and tumor suppressor mechanisms (Figure 2).

Several studies demonstrate the important role of miRNAs in regulating breast cancer cell proliferation. In breast cancer cell lines, miR-4472 has been reported to reduce *RGMA* mRNA levels, an effect associated with increased breast cancer cell proliferation and tumor progression [51]. Similarly, miR-92a-3p enhances proliferative activity through repression of the tumor suppressor *BTG2* [52]. Exosomal miR-155-5p promotes the activation of the NF-κB signaling pathway via suppressing *NDFIP1*, thereby increasing inflammatory cytokine secretion and enhancing breast cancer cell proliferation and tumor progression [53] (Figure 2).

In contrast, several miRNAs exert tumor-suppressive effects by repressing oncogenic drivers and limiting proliferative activity. For example, miR-362-3p has been shown to inhibit proliferation through direct interaction with *hERG* mRNA [54]. Moreover, in vitro and in vivo studies have demonstrated that miR-874-3p interacts with *VDAC1* mRNA, reducing its expression and suppressing breast cancer cell proliferation [55]. Similar effects have been observed for miR-379-5p, which targets *KIF4A* to inhibit tumor progression [56].

Further scientific works highlight the tumor-suppressive roles of miRNAs in breast cancer cells. Overexpression of miR-205-5p reduces *LRP6* expression in MDA-MB-231 and MCF-7 cell lines, accompanied by suppression of Wnt/β-catenin target genes and inhibition of cell proliferation and tumor progression. However, a direct interaction between miR-205-5p and *LRP6* mRNA has not been experimentally validated [57]. In contrast, miR-516a-3p has been reported to directly target *PYGO2*, resulting in suppression of Wnt/β-catenin signaling and inhibition of cell growth and EMT [58]. Similarly, miR-449c-5p suppresses cell proliferation through repression of *ERBB2* and subsequent inhibition of JAK/STAT signaling [59].

Additional tumor-suppressive miRNAs include miR-16-5p, which inhibits cell proliferation by targeting *ANLN* [60], and miR-214-3p, which suppresses proliferative activity by targeting *B7H3* while simultaneously improving the immune microenvironment in breast cancer [61]. In TNBC, miR-26b-5p also inhibits cell proliferation by repressing *MYCBP* [62]. Notably, miR-378 is frequently downregulated in breast cancer, and its reduced expression has been associated with enhanced proliferative and migratory capacity. This dysregulation is linked to transcriptional regulation by the transcription factor XBP1 [63], establishing the XBP1/miR-378 axis as an important regulatory mechanism in breast cancer progression.

### 3.2. Apoptosis

Apoptosis is a tightly regulated form of programmed cell death that is essential for maintaining tissue homeostasis and eliminating damaged or potentially harmful cells [64]. In breast cancer, dysregulation of apoptotic signaling pathways contributes to tumor initiation, disease progression, and resistance to therapy [65]. Accumulating evidence demonstrates that miRNAs are key regulators of apoptosis through their ability to target critical components of cell death pathways (Figure 2).

Modulation of PI3K/Akt signaling is a common mechanism by which miRNAs influence apoptosis in breast cancer. Studies in MDA-MB-231 and MCF-7 cells demonstrate that miR-641 suppresses *NUCKS1* and the anti-apoptotic gene *BCL2*, while indirectly upregulating the pro-apoptotic genes *BAX*, *CASP3*, and *CASP9*. The proposed mechanism of action indicates that miR-641 directly targets *NUCKS1* mRNA, inhibiting PI3K/Akt signaling and promoting apoptosis [66]. A similar pro-apoptotic effect has been reported for miR-944, which directly targets *SPP1* mRNA, leading to inhibition of the same survival pathway and enhanced apoptotic cell death in vitro [67]. In contrast, miR-548k promotes tumor progression by activating the PI3K/Akt pathway and suppressing apoptosis in MCF-7 cells. Although PTEN has been proposed as a downstream target of miR-548k based on their relative expression patterns in breast cancer samples, a direct miR-548k–PTEN interaction has not been experimentally validated [68].

Additional miRNAs regulate apoptosis by modulating alternative signaling pathways. In MDA-MB-468 cells, miR-21 inhibition significantly enhances apoptosis, consistent with its role in suppressing multiple pro-apoptotic targets, including FASLG, PDCD4, and PTEN [69]. Similarly, miR-216b directly targets *HK2* mRNA, leading to reduced HK2 expression and promoting autophagy-associated programmed cell death via inhibition of the mTOR signaling pathway in both in vitro and in vivo models [70]. Furthermore, miR-34a, a transcriptional target of *TP53*, has been associated with reduced *SIRT1* expression and the establishment of a positive feedback loop that enhances p53-mediated apoptosis in breast cancer cells. However, whether *SIRT1* is a direct target of miR-34a was not experimentally validated in this study [71]. Consistent with its tumor-suppressive role, exogenous administration of miR-26a in MCF-7 xenograft models has been shown to reduce the expression of the oncogenes *MTDH* and *EZH2*, both direct targets of miR-26a, resulting in enhanced apoptosis [72] (Figure 2).

In addition to these findings, several other miRNAs have been implicated in the negative regulation of apoptosis and promotion of tumor progression. Overexpression of miR-125b in MDA-MB-435, MDA-MB-231, and BT-474 cell lines suppresses apoptosis through targeting of the pro-apoptotic gene *BAK1* [73]. Similarly, overexpression of miR-221/222 in MCF-7 cells has been associated with reduced apoptotic activity and enhanced tumor progression through direct repression of the pro-apoptotic genes *PUMA* and *CASP3*, as well as *p27Kip1*, a key cyclin-dependent kinase inhibitor involved in G1/S cell cycle arrest [74].

### 3.3. Tumor Angiogenesis

Angiogenesis, the process of new blood vessel formation, is a critical process in the progression of multiple malignancies, including breast cancer [75,76,77]. This complex and tightly regulated process is regulated by multiple pro- and anti-angiogenic factors that collectively influence tumor growth and progression [78]. Several studies recognize miRNAs as critical regulators of angiogenesis, exerting either pro- or anti-angiogenic effects through direct targeting of angiogenic mediators, transcriptional regulators, and upstream signaling intermediates [78] (Figure 2).

Multiple scientific works demonstrate that miRNAs promote angiogenesis in breast cancer, underscoring their critical role in the regulation of cancer-related processes. For instance, the exogenous administration of miR-146a-5p in BT-474 cells results in the upregulation of CCNB1 and downregulation of CDKN1A–two molecules involved in cell cycle regulation–leading to enhanced endothelial cell proliferation and, consequently, the promotion of angiogenesis [79]. Similarly, overexpression of miR-526b or miR-655 in MCF-7 cells increases the expression of multiple angiogenic mediators, including VEGF-A, VEGF-C, VEGF-D, COX-2, and LYVE-1, promoting angiogenesis [80]. In BRCA1/2-associated breast cancer, the reduced expression of miR-573 and miR-578 has been reported to induce the formation of new blood vessels due to loss of repression of target genes involved in focal adhesion signaling, VEGF signaling, and HIF-1–related pathways [81]. Under hypoxic conditions, the transcription factor HIF-1 is activated, resulting in increased SNHG1 expression. SNHG1 inhibits the expression of miR-199a-3p, resulting in increased levels of its direct target *TFAM*, and promoting angiogenesis [82].

Several miRNAs exert anti-angiogenic effects by directly targeting key angiogenic regulators. In MDA-MB-231 cancer stem-like cells, elevated miR-204 levels suppress angiogenesis through downregulation of *VEGF-A* and *CTNNB1*, indicating inhibition of both angiogenic and Wnt/β-catenin signaling pathways. However, the direct molecular targets responsible for these effects have not been experimentally validated [83]. Another study has demonstrated that miR-140-5p directly binds to *VEGF-A* mRNA in MDA-MB-231 and MCF-7 breast cancer cell lines, resulting in reduced VEGF-A expression and inhibition of angiogenesis [84]. Also, miR-126 has been reported to suppress angiogenic activity in MCF-7 cells, accompanied by decreased VEGF-A levels; however, the molecular mechanism underlying this regulation has not been elucidated [85]. Furthermore, miR-195 inhibits angiogenesis by targeting *IRS1* mRNA, leading to suppression of downstream signaling pathways involved in vascular development [86] (Figure 2). In vitro and in vivo studies have shown that ZLM-7 positively regulates the expression of miR-212-3p, leading to post-transcriptional suppression of *SP1*. Concurrently, reduced *SP1* expression leads to decreased VEGF-A levels, thereby inhibiting angiogenesis [87]. Conversely, activation of PI3K/Akt signaling by von Willebrand factor (vWF) suppresses miR-205-5p, relieving repression of its target *VEGF-A* and promoting VEGF-dependent angiogenic proliferation in MDA-MB-231 and MCF-7 cells [88].

### 3.4. Cell Migration and Invasion

The first step of the metastatic cascade is invasion, during which tumor cells penetrate their basement membrane and migrate into the surrounding tissues [89]. Cancer cell migration and invasion represent critical steps in breast cancer progression, making the molecules involved in these processes particularly important to study. Increasing evidence demonstrates that miRNAs play crucial roles in controlling breast cancer cell motility and invasiveness by post-transcriptionally regulating genes involved in key signaling pathways like MAPK, STAT3, Wnt/β-catenin, and TGF-β, as well as epigenetic regulatory mechanisms (Figure 2).

Several studies have identified miRNAs that suppress migration and invasion by directly targeting genes involved in pro-invasive signaling. For instance, miR-1260b downregulation is associated with increased *CCDC134* expression and subsequent activation of MAPK signaling, thereby promoting migratory and invasive behavior; however, a direct interaction between miR-1260b and *CCDC134* mRNA has not been experimentally validated [90]. Moreover, miR-556-5p inhibits migration and invasion in MDA-MB-231 and MCF-7 breast cancer cells via its binding to *PTHLH* mRNA. Another key regulatory axis involves miR-142-5p, which targets *DNMT1* mRNA and decreases its expression, resulting in epigenetic changes and activation of the tumor suppressor *SERPINB5*. Notably, MKL-1 has been found to activate the transcription of miR-142-5p, creating a positive feedback loop that results in the suppression of migration and invasion in breast cancer [91] (Figure 2).

Additional miRNAs exert their tumor suppressive effects by modulating EMT-related genes. For instance, miR-337-3p targets *ESRP1*, an epithelial-specific splicing factor that regulates alternative splicing during EMT [92], reducing its expression and suppressing migration and invasion in breast cancer cell lines [93]. Likewise, miR-518a-5p suppresses migration and invasion through direct targeting of *ZEB2* mRNA [94], a key transcription factor that drives EMT and mesenchymal phenotypes [95]. In addition, miR-138-5p targets *RHBDD1* mRNA–a previously identified inducer of migration and invasion in breast cancer -, reducing its levels and inhibiting these processes in breast cancer cells [96]. In addition to the aforementioned miRNAs, miR-125b-5p has been shown to inhibit migration and invasion by targeting *KIAA1522* mRNA [97], an oncogene associated with tumor differentiation [98].

Other miRNAs inhibit migration and invasion by regulating key signaling pathways. MiR-135a-5p binds to *BAG3* mRNA, suppressing its expression and inhibiting proliferation, migration, and invasion. It is known that BAG3 is implicated in the activation of cell cycle as well as mTOR and TGF-β signaling pathways, highlighting its relevance in breast cancer aggressiveness [99]. In addition, miR-148a has been found to inhibit migration and invasion by targeting *WNT1* mRNA—a gene that encodes a ligand of the Wnt/β-catenin signaling pathway—leading to attenuation of Wnt/β-catenin signaling activity [100]. Of note, miR-548m has been shown to reverse EMT and reduce migratory and invasive capacities. Mechanistically, miR-548m overexpression increases E-cadherin levels while indirectly suppressing EMT-associated transcription factors, including SNAI1, SNAI2, ZEB1, and ZEB2. *AHR* was identified as a direct target of miR-548m, underscoring the crucial role of miRNAs within EMT-regulatory networks [101].

In contrast to these tumor-suppressive miRNAs, certain miRNAs have been reported to promote migration and invasion in breast cancer. In BT-474 and MDA-MB-231 cells, miR-30d targets *KLF11* mRNA, leading to reduced KLF11 expression and subsequent activation of the STAT3 signaling pathway, thus enhancing migratory and invasive behavior [102]. Likewise, miR-532-5p promotes proliferation and migration in MDA-MB-231 cells by binding to the mRNA of the growth inhibitor RERG, resulting in activation of the MAPK/ERK signaling pathway [103].

Emerging evidence has also highlighted the role of exosomal miRNAs in the regulation of invasive phenotypes. For example, exosomal miR-7-5p targets *RYK*, leading to reduced phosphorylation of JNK and c-Jun, key mediators of EMT-associated transcriptional programs. This signaling modulation suppresses EMT, migration, and invasion in breast cancer cells, underscoring the importance of intercellular miRNA transfer in modulating metastatic traits [104].

### 3.5. Metastatic Dissemination and Colonization

The dissemination of tumor cells from the primary site to distant anatomical sites accounts for the majority of cancer-related deaths. Metastasis is a multistep process that extends beyond local invasion and involves intravasation, survival in circulation, extravasation, and colonization of distant organs. These steps require dynamic phenotypic plasticity, including reversible EMT, resistance to anoikis and shear stress, immune evasion, metabolic adaptation, and the ability to establish and remodel the pre-metastatic niche [105] (Figure 2).

Accumulating evidence has identified miRNAs as critical regulators of metastatic progression in breast cancer, with widespread miRNA dysregulation representing a hallmark of metastasis. Several oncogenic miRNAs have been implicated in promoting tumor dissemination. For instance, miR-7641 is overexpressed in breast cancer, and it has been demonstrated to be transferred via exosomes, promoting tumor progression and metastatic spread [106]. In addition, in vitro and in vivo studies have shown that miR-223-3p promotes metastasis by targeting *FBXW7* mRNA, a tumor suppressor responsible for the degradation of oncogenic proteins involved in EMT and proliferation [107]. In MDA-MB-231 and T47D cells, miR-934 represses *PTEN*, resulting in activation of PI3K/Akt signaling and enhanced metastatic potential [108]. Furthermore, recent studies have demonstrated that exosomal miR-122-5p contributes to metastatic progression by targeting *MKP-2*, thereby sustaining MAPK pathway activation [109].

Metabolic reprogramming is an additional miRNA-regulated determinant of metastasis. Notably, miR-328-3p suppresses metastasis through targeting CPT1A, a key enzyme involved in fatty acid β-oxidation, thereby limiting the energy supply required for tumor progression [110]. Similarly, miR-4638-3p inhibits metastatic spread through repression of *ATF3*, while in vivo studies demonstrate that its overexpression significantly reduces bone metastasis in murine models of breast cancer [111]. In TNBC, miR-606 suppresses metastasis by targeting *STC1* mRNA, a hypoxia-responsive factor implicated in tumor cell survival and metastatic progression [112].

Several miRNAs regulate metastasis through transcriptional and inflammatory signaling networks. Studies have shown that miR-199a-5p targets *SP1*, suppressing its expression and consequently inhibiting the activation of *LOXL2*, a gene associated with EMT and metastasis. This regulatory axis is antagonized by TCF19, which downregulates miR-199a-5p, resulting in *LOXL2* activation and enhanced EMT and metastasis in breast cancer [113]. In TNBC, miR-206 has been demonstrated to disrupt actin dynamics and metastatic signaling by targeting *TWF1*. Downregulation of *TWF1* increases intracellular free G-actin levels, promoting sequestration of MKL1 and preventing its nuclear translocation and interaction with the transcription factor SRF. This, in turn, suppresses *IL11* transcription, which is required for EMT and metastasis [114]. The let-7/LIN28/NF-κB/IL-6 axis represents another important regulatory network involved in metastasis. In particular, let-7 suppresses *MYC* expression, whereas MYC induces *LIN28* expression, which in turn inhibits let-7 maturation, establishing a double-negative feedback loop [115]. Consequently, reduced let-7 levels result in increased *IL6* expression, which can either activate the JAK/STAT3 signaling pathway or reactivate NF-κB, thereby stabilizing the malignant phenotype and promoting metastasis [116].

Conversely, additional miRNAs have been implicated in the regulation of EMT-associated signaling pathways linked to metastasis. For example, miR-4435-2HG activates the JNK/c-Jun and p38 MAPK pathways, thereby promoting the EMT phenotype in TNBC [117]. In contrast, miR-93-5p and miR-520c exert anti-metastatic effects through direct targeting of MKL1 and STAT3, both key transcriptional mediators of EMT and inflammatory signaling, thereby inhibiting EMT and metastatic progression in breast cancer [118,119].

## 4. Strategies to Modulate miRNA Expression in Breast Cancer Research

Since dysregulated miRNA expression is widely recognized as a hallmark of cancer, substantial research efforts have been directed toward the development of strategies that enable precise and effective modulation of miRNAs [32,120]. These approaches are primarily based on either the restoration of tumor-suppressive miRNAs or the inhibition of oncogenic miRNAs, leveraging advances in nucleic acid delivery systems, chemical modifications, and genome engineering technologies. Beyond serving as valuable experimental tools for investigating cancer-associated molecular mechanisms, miRNA-targeting strategies hold considerable therapeutic potential. [14]. Through the controlled modulation of specific miRNAs, these approaches provide important insights into their biological roles and support the development of novel therapeutic interventions targeting dysregulated molecular pathways.

### 4.1. miRNA Replacement Approaches

The restoration of tumor-suppressor miRNAs that are downregulated or deleted in cancer is commonly referred to as miRNA replacement. This strategy has been extensively utilized in basic research as a gain-of-function approach to investigate the biological roles of specific miRNAs, enabling the identification of their target mRNAs and associated cancer-related pathways. By reintroducing functional miRNAs into cancer cells, researchers can evaluate their effects on key cellular processes, including proliferation, apoptosis, migration, invasion and tumor progression. Importantly, miRNA replacement also exhibits considerable therapeutic potential, as restored miRNAs can repress oncogenic targets, thereby inhibiting tumor growth and promoting programmed cell death [14].

Two principal strategies have been developed to achieve miRNA replacement: the delivery of synthetic miRNA mimics and the use of plasmid or viral vector expression systems encoding tumor-suppressive miRNAs [121].

#### 4.1.1. miRNA Mimics

**Figure 3:**
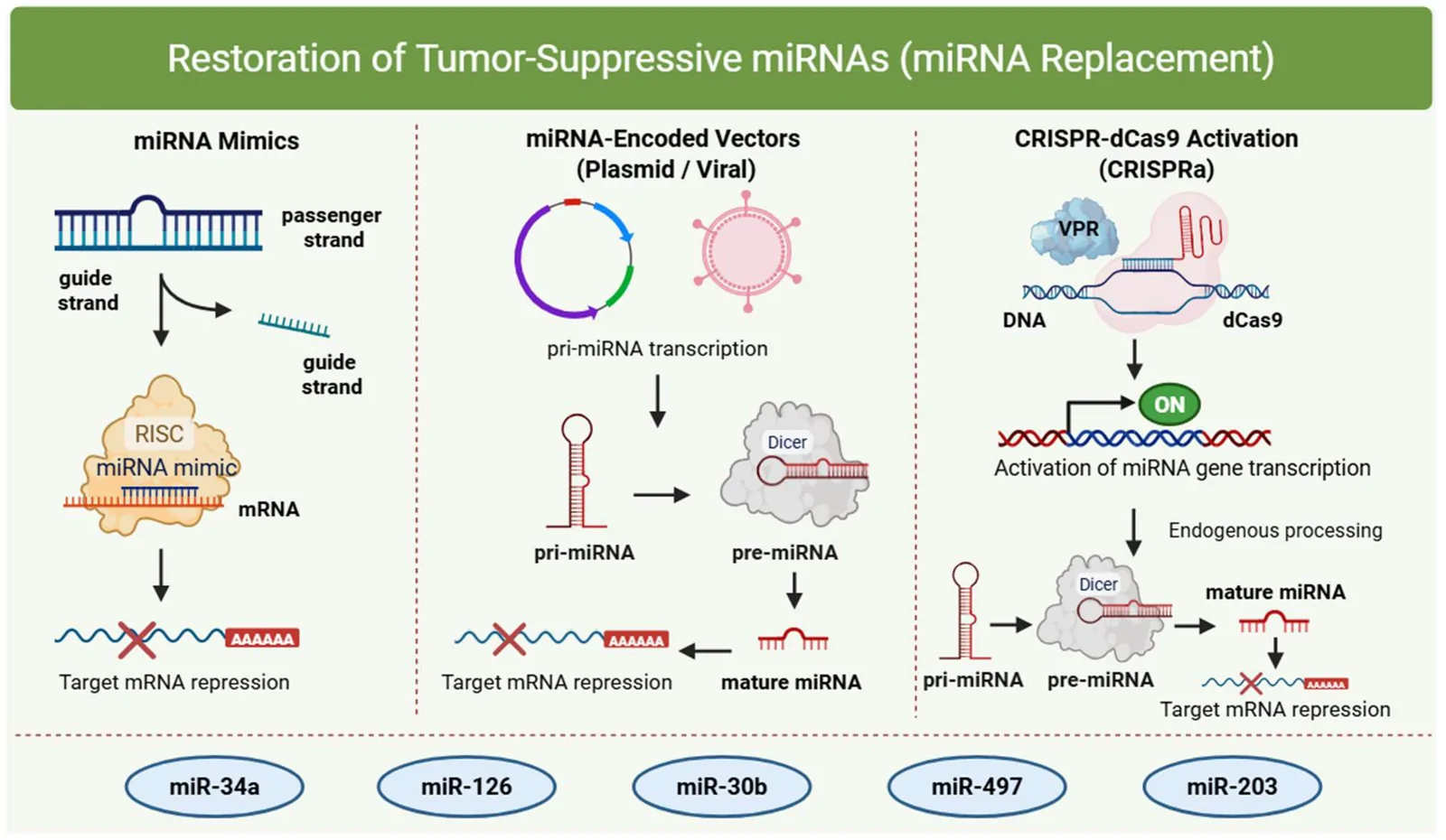
Restoration of miRNA expression. Schematic representation of miRNA mimics, miRNA-encoded vectors, and CRISPR activation (CRISPRa), which are used to restore or enhance miRNA expression for functional studies and therapeutic applications in breast cancer. Representative miRNAs investigated in breast cancer are shown.

miRNA mimics are synthetic double-stranded RNA oligonucleotides designed to imitate endogenous mature miRNAs in cells [122]. Structurally, they consist of a guide strand corresponding to the mature miRNA sequence and a passenger strand that is partially complementary to the guide strand. The passenger strand is typically chemically modified to enhance nuclease resistance and improve stability during delivery. These modifications also facilitate its exclusion from the RNA-induced silencing complex (RISC), enabling preferential incorporation of the guide strand, which mediates sequence-specific repression of the target mRNAs [122,123] (Figure 3).

The use of miRNA mimics has been extensively applied in breast cancer, primarily as a tool for investigating the biological roles of specific miRNAs. For example, the introduction of miR-641 mimics into breast cancer cells restores miR-641 levels, suppressing proliferation and promoting apoptosis through modulation of the NUCKS1/PI3K/AKT signaling pathway [66]. Similarly, exogenous administration of miR-337-3p mimics in MCF7 cells inhibits cellular migration and invasion [93], whereas restoration of miR-126 expression suppresses angiogenesis by directly targeting VEGF-A [85]. Additional studies have demonstrated that miR-653-5p mimics inhibit proliferation and migration [124], miR-16-5p mimics suppress cell proliferation [60], and miR-874-3p mimics reduce proliferation, migration, and invasion in breast cancer cells [55]. Together, these findings highlight the critical contribution of miRNAs to breast cancer development and progression, as well as the therapeutic potential of miRNA mimics for restoring tumor-suppressive miRNA activity.

#### 4.1.2. miRNA-Encoded Expression Vectors

An alternative miRNA replacement strategy involves the use of plasmids engineered to express precursor forms of endogenous miRNAs, including primary (pri-miRNAs) and precursor (pre-miRNAs) transcripts. These recombinant constructs are introduced into target cells either through non-viral transfection approaches or via viral delivery systems. Following expression, precursor miRNAs are processed through the endogenous miRNA biogenesis pathway to generate mature, functional miRNAs (Figure 3). This approach restores intracellular miRNA levels, enabling the regulation of target gene expression and the re-establishment of tumor-suppressive regulatory functions [125,126].

Although vector-based approaches are generally more complex and less commonly employed than synthetic miRNA mimics, they remain valuable tools in breast cancer research for elucidating miRNA function. For instance, plasmid-mediated restoration of miR-143 expression has been shown to inhibit migration and proliferation in breast cancer cells [127]. Similarly, reintroduction of miR-5096 via a plasmid vector encoding pri-miR-5096 suppresses proliferation and induces ferroptotic cell death in breast cancer models [128]. In addition, transfection of breast cancer cells with plasmids encoding miR-30b restores its expression and reduces proliferation, migration, and invasion [129]. Furthermore, lentiviral delivery of recombinant plasmids encoding miR-34a in TNBC cells has been reported to induce apoptosis and suppress cell proliferation [130].

While miRNA replacement strategies have significantly advanced both the functional characterization and therapeutic exploitation of tumor-suppressive miRNAs, complementary approaches focused on the inhibition of oncogenic miRNAs have also been used as critical tools for modulating dysregulated miRNA networks in cancer.

### 4.2. Strategies for miRNA Inhibition

**Figure 4:**
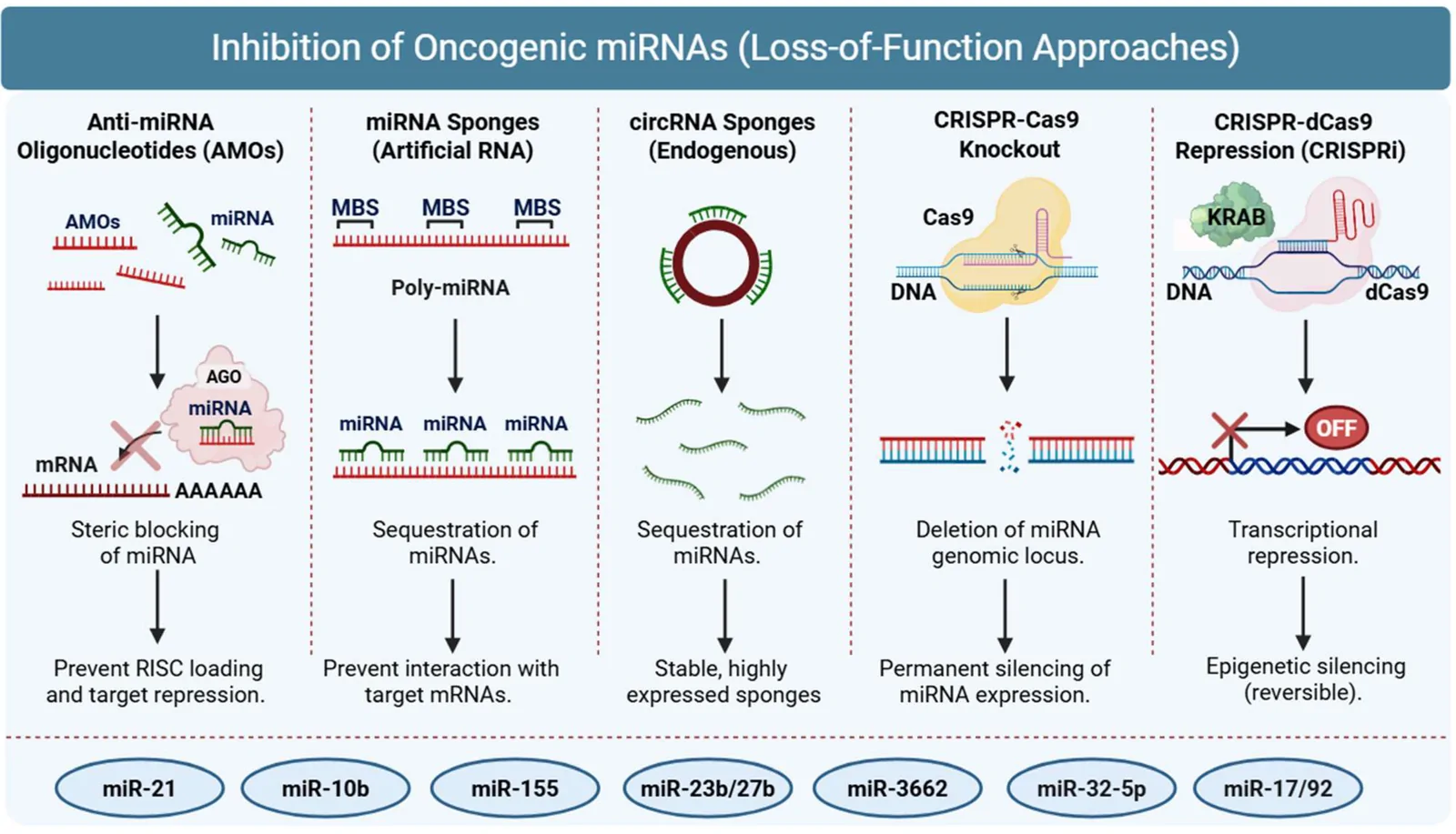
Strategies for miRNA inhibition. Major approaches for suppressing miRNA expression or activity, including anti-miRNA oligonucleotides (AMOs), miRNA sponges, circular RNA (circRNA) sponges, CRISPR-Cas9-mediated gene knockout, and CRISPR interference (CRISPRi). These strategies enable functional studies of miRNAs and are being explored for therapeutic applications in cancer. Representative miRNAs investigated in breast cancer are shown. AGO, Argonaute protein.

Strategies aimed at miRNA inhibition focus on suppressing oncogenic miRNAs (oncomiRs), which are frequently overexpressed in cancer. These approaches are widely employed in basic research as loss-of-function methods to investigate the biological roles of individual miRNAs (Figure 4). By selectively inhibiting specific miRNAs, researchers can identify their downstream target genes and associated signaling pathways, while also evaluating their contribution to key oncogenic processes, including proliferation, apoptosis, invasion, and metastasis.

Several approaches have been developed to achieve miRNA inhibition, including miRNA sponges, anti-miRNA oligonucleotides (AMOs), miRNA masking, CRISPR-Cas-based systems, and miRNA decoys. However, the application of miRNA masking and decoy-based strategies in breast cancer research remains relatively limited.

#### 4.2.1. miRNA Sponges

miRNA sponges are artificial RNA transcripts containing multiple repeated miRNA-binding sites (MBS) that mimic those present in endogenous target mRNAs (Figure 4). These binding sites are generally designed to complement the seed region of the mature miRNA, allowing the sponge to sequester either individual miRNAs or entire miRNA families sharing the same seed sequence. Upon expression, miRNA sponges function as competitive inhibitors that bind endogenous miRNAs and prevent their interaction with target mRNAs, thereby resulting in derepression of those target genes [131,132,133]. In addition, circular RNAs (circRNAs) have also been exploited as highly stable miRNA sponges for miRNA modulation, owing to their intrinsic stability and resistance to degradation [134,135].

miRNA sponge strategies have been extensively utilized in breast cancer research for functional characterization of oncogenic miRNAs. For instance, transfection of breast cancer cells with a miR-10b sponge inhibits miR-10b activity, resulting in upregulation of its target gene *HOXD10* and suppression of invasion and metastasis [136]. Similarly, a miRNA-21 sponge has been shown to reduce miR-21 activity, leading to decreased cancer cell viability and suppression of the cancer stem cell (CSC) phenotype [137]. miR-21 has also been targeted using circular RNA sponges. Notably, circ-21 has been reported to reduce miR-21 levels, leading to decreased cell migration, cell cycle arrest, and suppression of tumor progression. Furthermore, circ-21 was found to enhance the anticancer activity of doxorubicin by reducing the expression of drug resistance-associated genes [138].

More recently, advanced sponge designs have expanded the versatility of this approach. The use of a chimeric AXL-targeting aptamer–miR-214 sponge construct has been shown to inhibit migration and tumor progression. In this system, the sponge targets endogenous miR-214, while the aptamer component enhances delivery specificity by selectively directing the construct to target cells [139]. In addition, multivalent miRNA sponge systems capable of simultaneously suppressing multiple oncogenic miRNAs represent another promising tool for the modulation of miRNA activity. Synthetic sponge constructs targeting members of the miR-17/92 cluster have been reported to disrupt MYC-driven regulatory feedback loops, resulting in selective cytotoxicity in MYC-amplified breast cancer cells [140]. Other multivalent sponge systems have similarly demonstrated the ability to suppress tumor migration and progression through simultaneous targeting of multiple oncogenic miRNAs [141].

#### 4.2.2. Anti-miRNA Oligonucleotides

Anti-miRNA oligonucleotides (AMOs) constitute a specialized class of antisense oligonucleotides (ASOs) used for miRNA inhibition. AMOs are synthetic oligonucleotides designed to bind complementary sequences within mature miRNAs, thereby preventing their interaction with target mRNAs and their incorporation into the RISC [15] (Figure 4). Unlike classical ASOs, which typically induce RNase H-mediated degradation of target mRNAs, AMOs function through steric blocking of miRNA [142,143].

To improve their stability, specificity, and resistance to nuclease degradation, AMOs are frequently chemically modified. Common modifications include 2′-O-methyl (2′-OMe), 2′-O-methoxyethyl (2′-MOE), 2′-fluoro (2′-F), locked nucleic acids (LNA), phosphorothioate (PS) backbones, phosphorodiamidate morpholino oligomers (PMO), and peptide nucleic acids (PNA) [15,144,145,146]. These modifications significantly enhance their suitability for experimental and therapeutic applications.

The application of AMOs in breast cancer models has provided important insights into the functional roles of cancer-related miRNAs and their associated signaling pathways. For example, inhibition of miR-100 using AMOs increases the expression of its target gene *MTMR3*, promoting programmed cell death and suppressing tumor progression [147]. In addition, LNA-modified anti-miR-19-3p has been shown to reduce miR-19-3p levels, decrease cell viability, and enhance apoptosis, thereby suppressing tumor progression [148]. Furthermore, the delivery of LNA-antimiR-142-3p via exosomes reduces the expression levels of the oncogenic miR-142-3p and suppresses breast cancer stem cell tumorigenesis in vivo [149]. Similarly, inhibition of miR-32-5p using LNA-based AMOs results in upregulation of *FBXW7* and downregulation of *MYC*, leading to reduced proliferation and increased apoptosis [150]. Finally, targeting miR-148a with chemically modified AMOs increases *TXNIP* expression and suppresses proliferation of breast cancer cells [151].

#### 4.2.3. The Emerging CRISPR-Cas Gene Editing Systems

The discovery of the CRISPR-Cas system in 2010 marked a significant breakthrough in the field of genome engineering, providing a highly precise and versatile tool for targeted genetic manipulation [152]. Originally identified as part of the adaptive immune system of bacteria and archaea, CRISPR-Cas protects host organisms from foreign invaders like plasmids and viruses [153]. This defense mechanism operates through three phases: adaptation, expression, and interference. During adaptation, short fragments of foreign DNA, known as protospacers, are captured and integrated into the host’s CRISPR array by Cas1 and Cas2 proteins. During the expression phase, the CRISPR array is transcribed and processed into CRISPR RNAs (crRNAs). Finally, during interference, these guide RNAs direct Cas proteins to complementary nucleic acid sequences, resulting in target cleavage and degradation [16].

The genome-editing capacity of CRISPR-Cas systems derives from their ability to introduce double-stranded breaks into DNA, which are subsequently repaired via homologous recombination (HR) or non-homologous end joining (NHEJ). Multiple CRISPR-Cas systems have been identified, including Cas9, Cas12a and Cas12b, Cas13, and Cas14. All CRISPR-Cas platforms consist of two essential components: a guide RNA, typically engineered as single-guide RNA (sgRNA), and a native or engineered Cas effector protein [154,155,156]. Among these systems, CRISPR-Cas9 remains the most widely used platform for DNA editing, enabling gene knockout, correction, or replacement [157]. More recently, CRISPR-Cas13 has attracted increasing attention due to its ability to selectively target RNA rather than DNA, allowing transient and reversible gene regulation without permanent genomic alterations [15,158].

CRISPR-Cas technologies have emerged as powerful tools in cancer research and therapy, enabling the silencing of oncogenes and the restoration of tumor suppressive functions. Importantly, these systems can also be adapted to modulate miRNA expression and thereby regulate cancer-related pathways. In particular, CRISPR-Cas9 can disrupt genomic loci encoding oncogenic miRNAs, thereby effectively suppressing their expression, whereas CRISPR-Cas13 offers a more selective and reversible strategy for directly targeting mature miRNA transcripts at the RNA level [159].

Although the application of CRISPR-Cas systems in breast cancer research remains relatively limited, particularly regarding miRNA modulation, emerging studies provide promising proof-of-concept evidence. In vitro and in vivo studies have demonstrated that CRISPR-Cas9-mediated knockout of the genes encoding the oncogenic miR-23b and miR-27b suppresses tumor growth by significantly reducing cell proliferation and metastasis [160]. In addition, CRISPR-Cas9-mediated deletion of the miR-3662 genomic locus in TNBC cell lines reduces proliferation, impairs metastatic behavior, and suppresses tumor progression. Notably, transcriptional activation of miR-3662 using the CRISPR activation (CRISPRa) approach had the opposite effect, enhancing tumor growth and aggressiveness [161]. These findings are particularly important as they not only provide functional validation of miR-3662 in TNBC progression but also highlight the versatility of CRISPR-based platforms for both loss- and gain-of-function studies of miRNA activity. Beyond direct genome editing and RNA targeting, recent advances have further expanded CRISPR technology toward transcriptional and epigenetic regulation through the development of nuclease-deactivated Cas variants, which are discussed in the following section.

### 4.3. Epigenetic and Transcriptional Regulation

As discussed above, the conventional CRISPR-Cas9 systems primarily function through direct genome editing via the induction of double-strand breaks into DNA. Subsequent engineering of Cas9 has led to the development of a catalytically inactive variant, termed dead Cas9 (dCas9), and the corresponding CRISPR-dCas9 platform [162]. In contrast to active Cas9, dCas9 retains sequence-specific DNA-binding capacity while lacking endonuclease activity, thereby enabling modulation of gene expression without altering the underlying DNA sequence. This platform allows targeted activation or repression of genes through precise epigenetic and transcriptional regulation [163,164].

The nuclease activity of Cas9 depends primarily on the RuvC and HNH domains [165]. Introduction of point mutations into these domains inactivates endonuclease activity, while retaining its capacity for sequence-specific DNA binding directed by sgRNAs [16]. In this form, dCas9 can serve as a programmable DNA-binding scaffold that can be fused to a variety of effector domains in order to regulate gene expression at transcriptional and epigenetic levels.

Depending on the associated effector domain, CRISPR-dCas9 systems can either activate or repress gene expression. Transcriptional activation (CRISPRa) is typically achieved through fusion with activator domains like VP64, VPR, p300, MSK1, or TET1, whereas transcriptional repression (CRISPRi) is mediated by repressor domains including KRAB, DNMT3A, and histone deacetylases (HDACs) [163,166,167] (Figure 4). Through these fusions, CRISPR-dCas9 systems can induce targeted alterations of chromatin states, including DNA methylation and histone acetylation, thereby providing a powerful tool for precise and reversible control of gene expression.

A key advantage of CRISPR-dCas9 approaches lies in their ability to induce transient and non-permanent regulatory changes, making them particularly valuable for studying dynamic gene regulatory processes. These systems have also been adapted to investigate miRNA expression and function. A study published in 2026 described a CRISPR-dCas9-VPR system capable of selectively targeting cells undergoing EMT based on specific miRNA expression signatures, thus enabling elimination of pathogenic cell populations [168]. In addition, miRNA-activated CRISPR-dCas9-VPR systems have been engineered to be activated by specific miRNA signatures, thus triggering the activation of critical genes. Such designs enable selective modulation of disease-associated cellular processes or induction of targeted cell death, highlighting their potential as highly precise and programmable approaches for gene therapy [169].

The CRISPR-dCas9 technology has attracted increasing interest in cancer research because of its ability to repress oncogenic miRNAs or activate tumor-suppressor miRNAs without introducing permanent genomic alterations [167,170]. Its application in breast cancer, however, remains relatively limited due to the recent introduction of these technologies. Nevertheless, emerging studies provide important proof-of-concept evidence for their utility in modulating cancer-associated miRNAs. For example, reactivation of miR-200c using a CRISPR-dCas9-TET1 system suppresses oncogenic signaling pathways and inhibits tumor progression in breast cancer cells [171]. Conversely, in TNBC cell lines, epigenetic silencing of miR-3662 using CRISPR-dCas9-KRAB reduces tumor growth, whereas its activation via CRISPR-dCas9-VPR promotes proliferation and migration, enhancing tumor aggressiveness [161]. Together, these findings highlight the potential of CRISPR-dCas9 platforms as precise and versatile tools for investigating miRNA function and modulating miRNA expression through targeted epigenetic regulation, while avoiding permanent genomic alterations.

Despite the considerable potential of CRISPR-based technologies, several challenges must be addressed before their translation into clinical applications for breast cancer. A major limitation is the efficient and tumor-selective in vivo delivery of CRISPR components, including Cas proteins, sgRNAs, and associated regulatory elements. Current delivery systems face limitations related to tissue specificity, cargo capacity, immunogenicity, and potential toxicity, which limit therapeutic efficacy [172]. Off-target effects also represent a critical safety concern, as unintended recognition of genomic sequences by sgRNAs can result in undesired genetic alterations, potentially disrupting tumor suppressor pathways or activating oncogenic programs [173]. Although improved sgRNA design, high-fidelity Cas variants, and advanced computational prediction tools have enhanced editing specificity, comprehensive experimental validation remains essential to identify and minimize unintended modifications [174]. In addition, the bacterial origin of most Cas nucleases raises concerns regarding immunogenicity, as pre-existing humoral and cellular immune responses against commonly used Cas proteins, including SpCas9 and SaCas9, have been reported in humans [175]. Treatment-induced immune activation may further compromise the safety and efficacy of CRISPR-based therapeutics [176]. Therefore, the development of optimized delivery platforms, engineered Cas variants with reduced immunogenicity, and highly specific editing strategies will be critical for advancing CRISPR-based approaches toward safe and effective miRNA modulation in breast cancer.

## 5. miRNA-Based Therapeutics and Integration with Standard Breast Cancer Treatments

The idea of modulating miRNAs as a therapeutic approach in cancer has attracted considerable attention due to its potential to regulate complex oncogenic networks rather than individual molecular targets. Through the restoration of tumor-suppressive miRNAs or the inhibition of oncogenic miRNAs, these approaches have been proposed to simultaneously disrupt multiple processes involved in tumor progression, including proliferation, metastasis, and therapeutic resistance.

One of the most extensively investigated strategies involves miRNA replacement through the use of synthetic miRNA mimics. As discussed above, this approach aims to restore the physiological regulatory activity of tumor-suppressive miRNAs that are downregulated or lost in cancer. A representative example is miR-34a, a well-characterized tumor-suppressive miRNA that has been shown to regulate the expression of more than 30 oncogenes involved in multiple cancer-associated pathways [177,178,179,180,181,182]. Preclinical studies in breast cancer and other malignancies have demonstrated that restoration of miR-34a reduces cellular proliferation, invasion, and stemness, while simultaneously sensitizing tumor cells to chemotherapy [180,183]. These findings provided the basis for the development of MRX34, a liposomal miR-34a mimic that became the first miRNA-based therapeutic candidate to enter clinical trials. In a Phase I study involving patients with advanced solid tumors, including breast cancer patients, MRX34 administration was reported to be feasible and tolerable under oral dexamethasone premedication while preliminary antitumor activity was also observed. Although the trial was terminated due to immune-related adverse effects, the study presented a major milestone by demonstrating the feasibility of miRNA delivery in humans [184]. 

Beyond miR-34a, several additional miRNA mimics have demonstrated therapeutic potential in breast cancer xenograft mouse models. Independent studies have reported that restoration of miR-497 [185], miR-544 [186], miR-603 [187], and miR-203 levels exerts significant antitumor effects, [188], including suppression of tumor growth and inhibition of metastatic progression. These studies, conducted using miRNA mimics delivered via lipid-based nanoparticles or viral vectors, highlight the versatility of replacement strategies in targeting diverse oncogenic pathways (Table 2).

Conversely, miRNA inhibition strategies are designed to suppress oncogenic miRNAs that contribute to malignant progression. Approaches involving miRNA inhibitors like modified AMOs or miRNA sponges have demonstrated considerable potential to restore dysregulated gene expression and suppress tumor-promoting pathways [151,189]. Beyond their utility as experimental tools for investigating miRNA function, these strategies are increasingly being explored for therapeutic applications in breast cancer.

An important therapeutic application of miRNA modulation is its combination with established treatment modalities. Preclinical studies indicate that miRNA-targeting strategies can act synergistically with chemotherapy, radiotherapy, or targeted therapies, thereby enhancing therapeutic efficacy. For instance, a recent study demonstrates that the combination of AMOs targeting miR-21 and miR-155 with photodynamic therapy significantly suppresses proliferation and metastatic behavior in TNBC cells. In this approach, AMOs inhibit the oncogenic functions of miR-21 and miR-155, while the photosensitizer chlorin e6 (Ce6) generates reactive oxygen species (ROS) under light activation, inducing apoptosis of tumor cells [190]. In addition, miR-34a mimics have shown enhanced antitumor efficacy when combined with tyrosine kinase inhibitors such as sunitinib or cytotoxic drugs like adriamycin [180,191], likely owing to their capacity to simultaneously target multiple signaling pathways involved in drug resistance. In breast cancer xenograft models, restoration of miR-190 using mimics has been reported to enhance endocrine therapy sensitivity through modulation of the SOX9/Wnt/β-catenin axis [192]. In TNBC cell lines, miR-181a promotes resistance to PARP inhibitors and platinum-based therapy through *STING* targeting, whereas CMM489, a chemically modified miR-489 mimic, enhances PARP inhibitor sensitivity independently of *BRCA1/2* mutation status [193,194]. However, these latter findings remain limited to cell line models and require validation in appropriate in vivo models.

In HER2-positive breast cancer, miR-126 downregulation has been linked to trastuzumab resistance, whereas restoration of miR-126 levels has been shown to enhance drug sensitivity and improve treatment response. Consistent with these findings, several studies have reported improved trastuzumab efficacy when used in combination with miRNA mimics, including co-transfection with miR-26a and miR-30b mimics [195], as well as use of miR-126 or miR-770-5p mimics [196,197]. These observations further support the idea that miRNA-based interventions are particularly well suited for combination treatment aimed at overcoming therapeutic resistance.

Collectively, the available evidence supports miRNA modulation as a potential therapeutic approach in breast cancer (Table 2). However, the clinical translation of miRNA-based therapeutics remains limited by pharmacological, technological, and safety challenges. Naked miRNA molecules are inherently unstable and susceptible to nuclease-mediated degradation, while their physicochemical properties limit tissue penetration and efficient intracellular delivery. Following cellular uptake, insufficient endosomal escape can further restrict the amount of functional miRNA reaching the cytoplasm [198]. Consequently, therapeutic efficacy depends not only on selecting an appropriate miRNA target but also on achieving sufficient and sustained delivery to the relevant tumor cells.

Target specificity represents an additional challenge, as individual miRNAs can regulate multiple transcripts and signaling pathways, potentially affecting both intended and unintended molecular targets. For example, although *FBXO11* has been implicated as a target of miR-621 and may contribute to its effects on chemotherapy sensitivity, therapeutic restoration of miR-621 is unlikely to be restricted exclusively to the FBXO11/p53 axis [199]. Additional miR-621 targets may therefore contribute to its therapeutic effects as well as adverse effects. Furthermore, miRNAs are normally maintained at relatively low and tightly regulated intracellular levels, whereas therapeutic administration of miRNA mimics requires higher concentrations to achieve sufficient biological activity. Such high levels may increase interactions with lower-affinity or unintended transcripts, potentially reducing therapeutic specificity and increasing off-target effects.

Immunotoxicity represents another important barrier to the clinical development of miRNA therapeutics. Exogenous RNA molecules and their delivery systems can activate innate immune pathways and induce inflammatory responses, limiting therapeutic tolerability [198]. The clinical experience with MRX34 demonstrated that immune-related adverse events can substantially restrict the therapeutic window of systemically administered miRNA mimics, despite encouraging antitumor activity.

Taken together, these challenges highlight that successful clinical translation of miRNA-based therapies requires more than identifying miRNAs with antitumor activity. Future development should prioritize validated targets, tumor-selective delivery, appropriate dosing, and careful assessment of off-target and immune-related effects. Nevertheless, the potential to combine miRNA modulation with established therapies, particularly to enhance treatment response or overcome therapeutic resistance, provides a strong rationale for continued investigation and may support their future integration into personalized breast cancer treatment.

## 6. Advances in Delivery Platforms for miRNA Therapeutics

Achieving efficient and selective delivery of miRNA-based therapeutics to target tissues remains a major challenge due to the instability of RNA molecules and their high susceptibility to degradation [200]. Additional barriers, including limited cellular uptake, rapid clearance, and insufficient tissue specificity, further compromise therapeutic efficacy in vivo. Moreover, the potential induction of immune responses and off-target effects represents an additional obstacle for the safe clinical application of miRNA-based strategies. To address these limitations, a broad range of delivery platforms has been developed, which are generally classified into viral and non-viral systems. Continuous advances in vector engineering and nanotechnology have further contributed to the development of safer, more efficient, and more targeted delivery platforms [201]. In the following section, we discuss the major systems employed for miRNA delivery and their applications in breast cancer therapy, with particular emphasis on emerging technologies that have significantly improved delivery efficiency and therapeutic potential (Table 3).

### 6.1. Virus-Mediated Delivery Methods

Viral vectors were among the earliest systems developed for nucleic acid delivery and continue to represent powerful tools for gene transfer in cancer research. These systems ensure extremely high efficiency and sustained expression over time, making them promising strategies for replacing or repressing endogenous miRNAs [202]. The main types of viral vectors used for miRNA modulation include gamma-retroviral (RVs), lentiviral (LVs), adenoviral (Ads), adeno-associated viral vectors (AAVs), and herpes simplex virus (HSV)-based systems, each with its own unique characteristics, uses, and limitations.

#### 6.1.1. Retroviruses

Gamma-retroviral and lentiviral vectors, both members of the Retroviridae family, have the ability to integrate into the host’s genome, enabling stable and long-term miRNA expression. Gamma-retroviral vectors can transduce actively dividing cells, whereas lentiviral vectors can efficiently infect both dividing and non-dividing cells due to their capacity to traverse the nuclear membrane independently of mitosis [203]. This feature makes lentiviral vectors highly popular for preclinical and clinical applications, while limiting the utility of gamma-retroviruses for miRNA restoration into stem cells [204].

Despite their capacity for long-term expression, the use of integrating viral vectors is limited by the risk of insertional mutagenesis, which may disrupt genomic integrity of the host and potentially promote oncogenic transformation or other adverse effects [205,206]. Consequently, considerable efforts have focused on the development of safer expression constructs, including self-inactivating lentiviral vectors and integration-deficient systems designed to reduce genotoxicity while preserving delivery efficiency [203].

#### 6.1.2. Adenoviruses and Adeno-Associated Viruses

Adenoviral vectors were among the first viral delivery systems widely adopted for gene therapy applications and remain extensively used for transient transgene expression [207]. Owing to their relatively high cloning capacity, adenoviral and lentiviral vectors are preferred to co-express multiple miRNAs from a single polycistronic transcript [208,209]. Such polycistronic designs enable simultaneous delivery of several miRNA precursors to enhance gene silencing efficiency or to modulate complex regulatory networks. Adenoviral vectors are also capable of transducing a broad range of cell types, including both dividing and non-dividing cells [210].

A major drawback of these vectors is their high immunogenicity, a feature that limits their in vivo applicability despite their high transduction efficiency [202]. Adenoviral vectors are rendered replication-defective through deletion of essential genes, most commonly the E1 region required for viral replication. Consequently, recombinant adenoviruses must be produced in complementing packaging cell lines that provide E1 functions in trans [208]. Recombinant adenoviral vectors are capable of driving high levels of transgene expression. However, this expression is typically transient, often lasting only several days, due to the host’s immune responses against the viral components [209]. To address these limitations, second- and third-generation adenoviral vectors have been developed through additional deletion of viral coding regions involved in replication and host immune activation. These modifications reduced vector-associated immunogenicity, improved safety profiles, and prolonged the duration of transgene expression [211].

Among viral delivery systems, recombinant adeno-associated viral vectors (AAVs) have emerged as particularly promising platforms for miRNA therapeutics. AAVs are non-pathogenic, exhibit relatively low immunogenicity, efficiently transduce a broad range of cell types, and support prolonged transgene expression with minimal genomic integration [212,213]. Their limited packaging capacity (~4.8 kb) is generally sufficient for miRNA delivery applications, given the relatively small size of miRNA expression cassettes [202]. However, pre-existing immunity remains a major challenge for their clinical application, as prior natural exposure to different AAV serotypes can result in circulating neutralizing antibodies that reduce the vector’s efficacy. To address this issue, strategies like engineering capsid immunogenic epitopes or using less prevalent serotypes have been proposed [214,215].

#### 6.1.3. Herpes Simplex Virus 1 (HSV-1)

HSV-1 vectors represent an alternative platform for miRNA-based therapeutics and are distinguished by their exceptionally large cargo capacity, which enables the incorporation of large or multiple therapeutic constructs within a single vector system [216]. Compared with adenoviral systems, HSV-1 vectors offer a simpler design and greater flexibility for the delivery of complex genetic payloads, including the simultaneous expression of multiple miRNAs.

Earlier HSV-1 vectors were limited by cytotoxicity, inflammatory responses, and transient transgene expression caused by residual viral gene activity [217]. However, new-generation engineered vectors with silenced or deleted immediate-early genes have demonstrated markedly improved safety and more stable transgene expression. Importantly, HSV-1 genomes remain mainly episomal, thereby reducing the risk of insertional mutagenesis associated with integrating viral systems [216]. These properties have increased interest in HSV-1 vectors as potential platforms for the delivery of complex miRNA-based therapeutics in cancer research.

#### 6.1.4. Viral Delivery Approaches for miRNA Modulation in Breast Cancer Research

In breast cancer research, viral vectors have been extensively employed for the restoration of tumor-suppressive miRNAs or the inhibition of oncogenic miRNAs through stable or transient expression systems. Although the available literature is continuously expanding, the following subsection highlights some of the most representative studies illustrating the application of viral delivery platforms in miRNA-based breast cancer therapeutics. Accumulating preclinical evidence has demonstrated the considerable potential of these approaches in modulating tumor progression, metastatic behavior, and treatment response.

Among viral delivery platforms, lentiviral vectors have been widely utilized owing to their ability to integrate into the host genome and sustain long-term transgene expression. For instance, lentiviral-mediated restoration of the miR-34a/NOTCH1 axis significantly reduced breast cancer stemness and enhanced sensitivity to chemotherapeutic agents in nude mouse models, underscoring its therapeutic relevance in targeting cancer stem cell populations and overcoming treatment resistance [218]. Similarly, lentiviral delivery of anti-miR-29a suppressed tumorigenesis and metastatic progression in breast cancer xenograft mouse models with challenging conditions like type 2 diabetes [219]. Additional in vivo studies further support the tumor-suppressive roles of miRNAs delivered via lentiviral systems, including miR-100, which inhibits self-renewal of breast cancer stem-like cells and reduces tumor progression, and miR-99a, which suppresses breast cancer growth through direct targeting of FGFR3 [220,221].

Beyond conventional miRNA replacement or inhibition strategies, viral vectors have also been adapted for synthetic regulatory applications. In particular, adenoviral vectors incorporating miRNA response elements (MREs) into therapeutic constructs have enabled tumor-selective gene expression based on endogenous miRNA profiles. A notable example involves *TRAIL* expression regulated by let-7 and miR-122 response elements, allowing selective activation of apoptotic pathways in breast cancer cells while minimizing off-target toxicity [222]. These approaches further illustrate the versatility of viral systems for the development of more selective and targeted therapeutic strategies.

In parallel, delivery systems using recombinant AAV have also gained considerable attention due to their favorable safety profile, reduced immunogenicity, and capacity to support prolonged transgene expression. Notably, systemic administration of dual-targeted AAVs in multifocal breast cancer models has demonstrated efficient tumor targeting and sustained therapeutic gene expression across multiple lesions [223]. Furthermore, engineered AAV systems have been incorporated into combinatorial therapeutic strategies, including neoantigen vaccine delivery in combination with radiotherapy, resulting in enhanced antitumor immune responses, increased cytotoxic T-cell activity, and significant suppression of tumor progression in experimental breast cancer models [224].

Taken together, these studies demonstrate that viral vector-mediated miRNA delivery in breast cancer is progressively advancing toward more precise and clinically applicable therapies. In particular, several engineering approaches aimed at reducing immunogenicity, improving safety profiles, and enhancing targeting specificity have already demonstrated promising results, substantially increasing the therapeutic potential of lentiviral and AAV-based systems.

### 6.2. Non-Viral Delivery Methods

The limitations associated with virus-mediated delivery systems have driven the development of non-viral platforms aimed at improving safety while maintaining efficient nucleic acid delivery. These systems encompass a broad range of organic and inorganic nanocarriers designed for the transport of therapeutic nucleic acids, including miRNA mimics and inhibitors. Although non-viral systems generally exhibit lower transfection efficiency compared to viral systems, they offer several important advantages, including reduced immunogenicity and toxicity, improved biocompatibility, greater scalability and flexibility in cargo design [225,226]. Moreover, recent advances in nanotechnology and biomaterials engineering have further enhanced their stability, tissue specificity, and intracellular delivery, thereby supporting the expanding application of non-viral systems in miRNA-based therapeutics for breast cancer [227].

#### 6.2.1. Organic Nanoparticles

Organic nanoparticles constitute one of the most extensively investigated classes of non-viral delivery systems and have undergone considerable optimization in recent years. These carriers are composed of biocompatible organic materials like lipids and polymers, which enhance miRNA stability, protect therapeutic cargo from nuclease-mediated degradation, and facilitate cellular uptake [228]. In addition, advances in material design have further improved their loading capacity, targeting, and controlled release, making them promising platforms for miRNA delivery in breast cancer.

##### Lipid-Based Nanocarriers

Lipid-based nanocarriers represent one of the most advanced and extensively investigated non-viral platforms for nucleic acid delivery, including miRNA-based therapeutics. Their relatively low toxicity, favorable biocompatibility, and capacity for large-scale manufacturing and sterilization have contributed to their broad application in biomedical research and their considerable translational potential for clinical use [229,230,231]. Among the available lipid-based delivery platforms, liposomes and lipid nanoparticles (LNPs) are widely used for RNA delivery because of their favorable physicochemical and biological properties [230,232].

Conventional liposomes are spherical vesicles composed of one or more lipid bilayers surrounding an aqueous core, a structure that enables the efficient encapsulation of hydrophilic molecules like miRNAs [231,233]. In contrast, LNPs are structurally distinct nanocarriers that typically consist of ionizable lipids, structural phospholipids, cholesterol, and polyethylene glycol (PEG)-conjugated lipids that self-assemble into a non-lamellar lipid matrix rather than a classical bilayer vesicle [234]. This unique composition enhances particle stability, facilitates endosomal escape, and improves intracellular delivery of nucleic acids.

A variety of neutral lipids, including dioleoylphosphatidylethanolamine (DOPE), 1,2-distearoyl-sn-glycero-3-phosphocholine (DSPC), 1,2-oleoyl-sn-glycero-3-phosphocholine (DOPC), and phosphatidylcholine (PC), have been employed for the development of stable lipid-based formulations with high miRNA loading capacity [235]. In parallel, cationic liposomal nanoparticles, commonly referred to as lipoplexes, have been extensively investigated due to their ability to electrostatically interact with negatively charged miRNAs and cellular membranes, thereby facilitating intracellular delivery and cellular uptake. Commonly used cationic lipids, such as DOTAP, promote efficient miRNA condensation and delivery. Nevertheless, cationic formulations are often associated with increased cytotoxicity, rapid clearance, and higher immunogenicity relative to neutral lipids.

Importantly, an increasing number of preclinical studies have demonstrated the therapeutic potential of lipid-based nanocarriers for miRNA delivery in breast cancer in vivo. For instance, liposome-mediated delivery of miR-6838-5p mimics in a nude mouse xenograft model resulted in significant suppression of tumor growth, which was associated with downregulation of the oncogene DDR1, highlighting the functional relevance of miRNA restoration in vivo [236]. Similarly, co-delivery approaches have shown substantial therapeutic promise. Co-encapsulation of miR-451 mimic together with a PARP1 inhibitor in cationic liposomes significantly inhibited tumor growth in BRCA1-deficient breast cancer xenografts through modulation of the PI3K signaling pathway and DNA damage response [237]. Furthermore, in HER2-positive breast cancer xenograft models, immunoliposomal systems co-delivering chemotherapeutic agents and miRNA inhibitors exhibited pronounced anti-tumor activity, including reduced cellular proliferation and enhanced apoptosis, while maintaining a favorable safety profile [238].

Additional studies further support the translational relevance of lipid-based miRNA delivery systems, particularly in aggressive breast cancer subtypes. Administration of miR-22-3p encapsulated in single-lipid liposomal nanoparticles (SLNPs) markedly suppressed tumor growth in orthotopic TNBC mouse models through inhibition of eEF2K and downstream PI3K/Akt and Src signaling pathways [239]. A similar strategy using miR-603 mimics encapsulated in DMPC/DSPE-PEG2000 nanoparticles also demonstrated significant antitumor activity in TNBC xenografts, where restoration of miR-603 expression reduced eEF2K levels and inhibited several oncogenic mediators, including Src, Akt, cyclin D1, and MYC [187]. Taken together, these findings highlight the capacity of lipid-based nanocarriers to efficiently deliver miRNAs in vivo to regulate critical oncogenic networks in breast cancer, supporting their continued development as promising therapeutic strategies.

##### Emerging Polymeric Nanoparticles

Polymeric nanoparticles represent an advanced class of delivery systems for nucleic acid therapeutics. These carriers are commonly composed of biodegradable and biocompatible polymers, including poly(lactic-co-glycolic acid) (PLGA) and polyethyleneimine (PEI), which are widely utilized for the encapsulation and delivery of miRNA-based therapeutics [228,240]. A key advantage of polymeric nanoparticles lies in their ability to protect miRNA molecules from enzymatic degradation during circulation, while their surfaces can be readily modified with targeting ligands to improve selective delivery to specific cell populations [228,241]. In addition, certain cationic polymers, particularly PEI, exhibit the so-called “proton sponge effect,” which promotes endosomal escape through osmotic swelling and subsequent disruption of endosomal membranes, thereby facilitating the release of the therapeutic cargo into the cytoplasm [228]. These features have established polymeric nanoparticles as highly versatile and customizable platforms for miRNA delivery.

Consistent with these advantages, several in vivo studies have demonstrated the efficacy of polymeric nanoparticles for modulating miRNA expression in breast cancer models. For example, a hyaluronic acid-targeted PEG-PLGA-coated mesoporous silica nanoparticle system co-delivering a miR-34a mimic and anti-miR-10b achieved efficient tumor targeting in TNBC models, resulting in significant inhibition of tumor growth and metastasis [242]. Similarly, a MUC1 aptamer-functionalized PLGA/PβAE nanocomplex co-delivering antimiR-21 and epirubicin exhibited enhanced tumor selectivity and greater antitumor efficacy in vivo compared with chemotherapy alone, highlighting the therapeutic benefit of combining miRNA modulation with conventional anticancer agents [243].

Additional studies further demonstrate that polymeric nanoparticles are particularly suitable for multi-targeted therapeutic approaches. Co-delivery of anti-miR-21 and anti-miR-10b using uPAR-targeted PLGA-b-PEG nanoparticles resulted in substantial inhibition of tumor growth in TNBC xenografts, even at low doses, underscoring the effectiveness of simultaneous targeting of multiple oncogenic miRNAs [244]. In another strategy, redox-responsive PEI-based nanoparticles (PEI-SPDP-Man) enabled efficient intracellular release of miR-34a in orthotopic TNBC models, leading to reduced tumor growth and metastasis through modulation of Bcl-2 and CD44 expression [245].

A key advantage of polymer-based systems is their capacity to incorporate tumor microenvironment-responsive mechanisms that enhance delivery specificity and therapeutic efficacy. For instance, MMP2-sensitive PEGylated β-cyclodextrin–PEI nanoparticles achieved enhanced tumor accumulation and improved therapeutic efficacy by enabling enzyme-triggered activation specifically at tumor sites [246]. Similarly, dextrin-modified PEI nanoparticles co-delivering miR-34a and CXCR4 antagonists demonstrated significant antitumor and anti-metastatic effects in both orthotopic and metastatic breast cancer models by simultaneously targeting multiple pathways involved in tumor progression [247].

Of note, hybrid platforms integrating polymeric materials with inorganic nanostructures have further expanded the functional capabilities of these delivery systems. For example, layered double hydroxide (LDH)-based nanocarriers delivering miR-30a significantly suppressed tumor growth in vivo without evident systemic toxicity, illustrating how polymer-assisted nanostructures can further enhance miRNA stability, delivery efficiency, and therapeutic performance [248].

##### The Innovative Plant-Derived Exosome-like Nanovesicles

Plant-derived exosome-like nanovesicles (PELNVs) are nanosized extracellular vesicles (approximately 50–150 nm) naturally secreted by plant cells. Although their biogenesis has not been fully elucidated, current evidence indicates that PELNVs can arise through multiple secretion pathways. The best characterized mechanism involves the formation of multivesicular bodies (MVBs), which fuse with the plasma membrane to release intraluminal vesicles into the extracellular space [249]. However, alternative biogenesis routes have also been proposed, including the exo-positive organelle (EXPO) pathway and vacuolar release, with additional evidence suggesting a potential contribution of autophagy-related mechanisms [250].

Structurally similar to mammalian exosomes, PELNVs consist of a phospholipid bilayer and contain diverse bioactive cargo, including nucleic acids, proteins, lipids, and metabolites that reflect their cellular origin. Compared with mammalian extracellular vesicles, PELNVs exhibit low immunogenicity, high biocompatibility, favorable stability, and minimal toxicity, making them attractive for biomedical and drug delivery applications [251]. Their natural composition also contributes to prolonged circulation and improved bioavailability [252].

Beyond their intrinsic biological activity, PELNVs are being investigated as potential delivery platforms for therapeutic agents [252,253]. In breast cancer research, they are emerging as promising nanocarriers for miRNA-based therapies, particularly in aggressive subtypes. Recent evidence demonstrates that exosome-like nanovesicles derived from Brucea javanica (BF-Exos) can deliver multiple functional miRNAs into breast cancer cells. In TNBC mouse models, these vesicles significantly suppressed tumor growth, angiogenesis, and metastasis by regulating key oncogenic pathways, while maintaining a favorable safety profile [254]. Additional evidence further supports the therapeutic potential of plant-derived nanovesicles in breast cancer. Exosome-like nanovesicles derived from grape callus cultures (GCENs) demonstrate selective anticancer activity in vitro by reducing the viability of TNBC MDA-MB-231 cells in a dose-dependent manner, while exhibiting minimal cytotoxicity toward non-cancerous HEK293 cells [255]. Notably, both BF-Exos and GCENs were evaluated based on their endogenous cargo, whereas the use of engineered PELNVs for the delivery of specific miRNA mimics or inhibitors remains at an early stage of development.

Despite these advances, several challenges currently limit the clinical translation of PELNVs as miRNA delivery systems. Although PELNVs can be engineered to encapsulate exogenous therapeutic cargo, including miRNAs, siRNAs, DNA, proteins, and small-molecule drugs, efficient and reproducible cargo loading remains challenging [249,250]. Current loading strategies, including passive incubation and active approaches such as sonication, electroporation, and extrusion, show variable loading efficiencies depending on both the physicochemical properties of the cargo and the loading method, while some techniques may compromise vesicle integrity [250]. In addition, PELNVs are inherently heterogeneous, with vesicle size, molecular composition, and drug-loading capacity varying according to plant species, tissue source, cultivation conditions, and isolation protocols, resulting in batch-to-batch variability [249]. Their complex endogenous cargo, comprising RNAs, proteins, lipids, and secondary metabolites, may further influence therapeutic outcomes and complicate attribution of biological effects to the delivered miRNA alone. Finally, limited pharmacokinetic characterization, incomplete safety evaluation, and the lack of standardized manufacturing and quality-control protocols remain important barriers to clinical translation [250]. Future efforts should therefore focus on improving cargo-loading strategies, engineering more uniform vesicle populations, and establishing standardized production and characterization methods to facilitate the development of reliable PELNV-based miRNA therapeutics.

#### 6.2.2. Inorganic Nanoparticles

Inorganic nanomaterials, including gold nanoparticles, silicon nanoparticles, quantum dots, and carbon nanotubes, represent alternative platforms for miRNA delivery. Compared with organic carriers, these nanoparticles exhibit distinct structural and physicochemical properties, while still allowing extensive surface functionalization despite their relatively low molecular weight [256]. Their main advantages include high structural stability, precise control over size, shape, and porosity during synthesis, efficient cargo-loading capacity, and straightforward conjugation with targeting ligands, making them highly attractive methods for nucleic acid delivery applications [257].

Among the inorganic nanomaterials investigated for miRNA delivery, gold nanoparticles (AuNPs) represent the most extensively studied platform in breast cancer research. Their widespread application is largely attributed to their favorable physicochemical and biological properties, including adjustable size and morphology, ease of surface modification, and high biocompatibility. In particular, surface engineering approaches involving PEG conjugation or biomimetic membrane coating have substantially improved nanoparticle stability, circulation time, and targeting capacity [258,259]. These features make AuNPs suitable carriers for overcoming major limitations associated with miRNA therapeutics, including molecular instability, nuclease-mediated degradation, limited bioavailability, and inefficient cellular uptake.

The therapeutic potential of AuNP-mediated miRNA delivery has been demonstrated in several preclinical breast cancer studies. For example, AuNPs have been successfully utilized for the delivery of miR-206 mimics in luminal A breast cancer cells, resulting in suppression of tumor progression through inhibition of the NOTCH3 signaling pathway [260]. In addition to their therapeutic efficacy, several studies have highlighted the favorable safety profile of these systems in vivo. Gold nanoparticle-mediated delivery of miR-708 mimics in TNBC mouse models has demonstrated significant antitumor effects without evidence of systemic toxicity. Histological examination revealed no morphological abnormalities in major organs, including the heart, liver, kidneys, spleen, and bone marrow, while hematological and serum biochemical analyses confirmed the absence of hepatic, renal, or muscular toxicity following treatment [261]. Similarly, multifunctional AuNPs co-delivering doxorubicin and a miR-21 inhibitor exhibited enhanced antitumor efficacy, further underscoring the therapeutic potential of combining miRNA modulation with conventional chemotherapeutic agents [262]. Importantly, no apparent toxic side effects, significant body weight changes, or treatment-related mortality were observed during the in vivo study period, further supporting the suitability and translational potential of these nanoparticle-based delivery systems.

## 7. Future Perspectives: From Network Modulation to Precision Oncology

The increasing knowledge of miRNA biology in breast cancer is about to reshape the framework of cancer therapeutics, shifting the focus from targeting a single gene toward network-level modulation. Given that individual miRNAs can regulate a great number of transcripts simultaneously, their therapeutic manipulation offers a unique opportunity to reprogram entire oncogenic pathways rather than inhibiting isolated molecular targets. This system-level approach is particularly relevant in breast cancer, where high molecular heterogeneity and adaptive resistance mechanisms frequently limit the long-term efficacy of conventional therapies.

Looking ahead, one of the major priorities in the field will be the identification and validation of subtype-specific miRNA regulatory networks across breast cancer. Integrating multi-omics approaches, including transcriptomic, epigenomic, proteomic, and metabolomic datasets, will be essential for reconstructing these networks and defining their biological and clinical significance in breast cancer. Such efforts may facilitate the development of predictive models capable of linking miRNA expression patterns with therapeutic response, disease progression, and clinical outcome. For these purposes, artificial intelligence and machine learning approaches are expected to play a central role in deciphering complex miRNA-mRNA interactions, as well as in identifying molecular signatures with potential clinical relevance.

Another important direction involves the improvement of miRNA-based therapeutic strategies. Although current approaches, including miRNA mimics and anti-miRNA oligonucleotides, have demonstrated considerable promise in preclinical models, their clinical translation is hindered due to challenges related to delivery, stability, and off-target effects. Advances in nanotechnology and targeted delivery systems are expected to significantly improve tissue specificity and minimize toxicity, enabling safer and more effective in vivo applications. Importantly, the development of novel therapeutic strategies combining miRNA modulation with chemotherapy, immunotherapy, endocrine therapy, or targeted agents may provide a more effective approach for overcoming therapeutic resistance and improving treatment efficacy.

Emerging genome engineering technologies, particularly CRISPR-based systems, are also expected to significantly advance the field of miRNA-targeted therapeutics. The ability to precisely and reversibly modulate miRNA expression at the transcriptional or epigenetic level offers unprecedented opportunities for the manipulation of cancer-associated regulatory networks. Future studies will likely focus on programmable, miRNA-responsive systems capable of selectively targeting cancer cells based on specific molecular signatures, thereby improving therapeutic effectiveness and reducing toxicity to normal tissues.

## 8. Conclusions

In summary, the future of miRNA research in breast cancer is likely to be characterized by a progressive transition from the study of individual miRNAs toward the reconstruction and analysis of complex miRNA-centered regulatory networks. Achieving this objective will require not only the development of more precise experimental and computational tools, but also closer integration between basic research and clinical practice. Ultimately, the successful incorporation of miRNA biology into precision oncology frameworks may enable more accurate disease classification, while facilitating the development and implementation of personalized therapeutic strategies.

## Figures and Tables

**Table 1:** Representative miRNA expression patterns across breast cancer molecular subtypes and biological sample types. The table summarizes representative miRNAs reported to be differentially expressed across luminal A, luminal B, HER2-positive, and TNBC subtypes, based on analyses of patient-derived tumor tissues, serum samples, or established breast cancer cell lines. The listed miRNAs reflect findings reported in the cited studies and are not intended to represent clinically validated subtype-specific biomarkers. Differences in sample type and experimental model should be considered when interpreting miRNA expression patterns across studies.

Breast Cancer Subtype	Sample Type	Representative Dysregulated miRNAs	Reported Biological Relevance
Luminal A	Tumor tissue	miR-30c-5p, miR-30b-5p, miR-99a/let-7c/miR-125b, miR-29a, miR-181a, miR-652	Regulation of proliferation, migration, hormone-related signaling
Luminal A	Serum	miR-29a, miR-181a, miR-652	
Luminal B	Tumor tissue	miR-182-5p, miR-200b-3p, miR-15b-3p, miR-149-5p, miR-193b-3p, miR-342-3p/5p	Increased proliferative and aggressive phenotype
HER2-positive	Tumor tissue	miR-125b, miR-4728-3p, miR-146a-5p, miR-181d, miR-195-5p	HER2-associated signaling, proliferation, therapeutic resistance
TNBC	Tumor tissue	miR-17-5p, miR-20a-5p, miR-92a-3p, miR-106b-5p	Proliferation, invasion, EMT
TNBC	Cell lines	miR-138-5p, miR-4324, miR-4800-3p, miR-6836-3p, miR-363-5p, miR-182-5p, miR-141-3p, miR-339-5p, miR-4655-3p, miR-4784, miR-664b-5p, and miR-6787-5p	

**Table 2:** Representative miRNA-based therapeutic strategies evaluated in breast cancer models. Summary of selected studies investigating miRNA replacement and miRNA inhibition strategies in breast cancer. The table highlights the miRNA involved, breast cancer subtype or experimental model, delivery platform, molecular targets or signaling pathways affected, and the resulting therapeutic outcomes. Emphasis is placed on studies demonstrating in vivo efficacy, including tumor growth suppression, inhibition of metastasis, induction of apoptosis, and enhanced sensitivity to conventional therapies.

miRNA Strategy	miRNA	Breast Cancer Subtype/Model	Delivery System	Main Molecular Target/Pathway	Therapeutic Outcome	In Vivo Validation
miRNA replacement	miR-34a mimic	Luminal A and TNBC	Lentiviral vectors, lipid nanoparticles, and polymeric nanoparticles	BCL2, CD44, NOTCH1	Reduced proliferation and metastasis	Yes
miRNA replacement	miR-22-3p mimic	TNBC	Single-lipid liposomal nanoparticles	eEF2K/PI3K-Akt/Src	Tumor suppression	Yes
miRNA replacement	miR-603 mimic	TNBC	DMPC/DSPE-PEG2000 nanoparticles	eEF2K, MYC	Reduced tumor growth	Yes
miRNA replacement	miR-206 mimic	Luminal A	Gold nanoparticles	NOTCH3	Suppressed tumor progression	In vitro
miRNA replacement	miR-708 mimic	TNBC	Gold nanoparticles	Metastasis-related pathways	Reduced tumor growth with low toxicity	Yes
miRNA inhibition	anti-miR-29a	TNBC with type 2 diabetes	Lentiviral delivery	SIRTUIN 1	Suppressed tumorigenesis and metastatic progression	Yes
Combined inhibition	anti-miR-21 + anti-miR-10b	TNBC	uPAR-targeted PLGA-b-PEG nanoparticles	Anti-apoptotic & metastatic pathways	Strong tumor suppression	Yes
Combined therapy	anti-miR-21 + epirubicin	TNBC	PLGA/PβAE nanoparticles	PTEN-associated signaling	Reduced proliferation	Yes
Combined therapy	miR-451 + PARP1 inhibitor	BRCA1-deficient BC	Cationic liposomes	PI3K + DNA damage response	Enhanced therapeutic efficacy	Yes
Combined therapy	miR-21 inhibitor + doxorubicin	Breast cancer	AuNPs	Chemoresistance pathways	Enhanced chemotherapy response	Yes

**Table 3:** Comparative overview of miRNA delivery platforms investigated in breast cancer. Comparison of the principal delivery systems currently discussed for miRNA therapeutics in breast cancer, including lipid-based nanoparticles, polymeric nanoparticles, inorganic nanomaterials, exosomes, plant-derived nanovesicles, and viral vector systems. The table summarizes the major advantages, limitations, functional modifications, and representative applications of each platform, together with their relative translational potential. Particular emphasis is placed on properties relevant to miRNA delivery, including cargo protection, targeting capability, endosomal escape, biocompatibility, and multifunctionality.

Delivery Platform	Main Advantages	Main Limitations	Modifications	Representative Applications in Breast Cancer
Lipid nanoparticles (LNPs)	High nucleic acid encapsulation efficiency, efficient intracellular delivery, scalable manufacturing, clinical translation	Immunogenicity, rapid clearance, limited stability	PEGylation, cationic lipids, immunoliposomes	miR-22-3p, miR-603, miR-451 delivery
Polymeric nanoparticles	Structural tunability, controlled release, multifunctionality	Variable toxicity, endosomal escape barriers	Aptamers, pH/redox-sensitive systems, PEG coatings	miR-34a, anti-miR-21, anti-miR-10b
Inorganic nanoparticles (AuNPs, etc.)	High stability, easy surface functionalization, imaging compatibility	Long-term biocompatibility concerns	PEGylation, biomimetic membranes	miR-206, miR-708, miR-21 inhibitor delivery
Plant-derived exosome-like nanovesicles	Low immunogenicity, natural origin, oral administration potential	Limited studies, standardization challenges	Natural miRNA cargo enrichment	BF-Exos in TNBC
Viral vectors	High transfection efficiency	Safety concerns, immunogenicity	Tissue-specific promoters	Lentiviral miR-34a delivery
CRISPR-Cas systems	Precise and programmable regulation	Delivery complexity, off-target concerns	dCas9 activators/repressors	miR-3662 modulation

## Data Availability

No new data were created or analyzed in this study. Data sharing is not applicable to this article.

## References

[1] Liu X., Miao W., Huang M., Li L., Dai X., Wang Y. (2019). Elevated Hexokinase II Expression Confers Acquired Resistance to 4-Hydroxytamoxifen in Breast Cancer Cells. Mol. Cell. Proteom..

[2] Gautam S., Maurya R., Vikal A., Patel P., Thakur S., Singh A., Gupta G.D., Kurmi B.D. (2025). Understanding drug resistance in breast cancer: Mechanisms and emerging therapeutic strategies. Med. Drug Discov..

[3] Saxena R., Chakrapani B., Sarath Krishnan M.P., Gupta A., Gupta S., Das J., Gupta S.C., Mirza A.A., Rao S., Goyal B. (2023). Next generation sequencing uncovers multiple miRNAs associated molecular targets in gallbladder cancer patients. Sci. Rep..

[4] O’Brien J., Hayder H., Zayed Y., Peng C. (2018). Overview of MicroRNA Biogenesis, Mechanisms of Actions, and Circulation. Front. Endocrinol..

[5] Wen K.-C., Sung P.-L., Yen M.-S., Chuang C.-M., Liou W.-S., Wang P.-H. (2013). MicroRNAs regulate several functions of normal tissues and malignancies. Taiwan. J. Obstet. Gynecol..

[6] Peng Y., Croce C.M. (2016). The role of MicroRNAs in human cancer. Signal Transduct. Target. Ther..

[7] Ghavi Dorabad D., Foruzandeh Z., Torki Z., Ebrahimi A., Hashemi S., Alivand M.R. (2024). Identification of dysregulated miRNAs and their roles in breast cancer; An in silico meta-analysis study. Inform. Med. Unlocked.

[8] Baylie T., Kasaw M., Getinet M., Getie G., Jemal M., Nigatu A., Ahmed H., Bogale M. (2024). The role of miRNAs as biomarkers in breast cancer. Front. Oncol..

[9] Ting J.Z.S., Lim J., Cho T.Y.Y., Yeoh Z.M., Yeo W.W.Y., Lim H.Y. (2026). Therapeutic potential of MicroRNAs in targeting breast cancer stem cells: A systematic review. Crit. Rev. Oncol..

[10] Pouya F.D., Rasmi Y., Gazouli M., Zografos E., Nemati M. (2022). MicroRNAs as therapeutic targets in breast cancer metastasis. Drug Deliv. Transl. Res..

[11] Papageorgiou S.G., Diamantopoulos M.A., Kontos C.K., Bouchla A., Vasilatou D., Bazani E., Scorilas A., Pappa V. (2019). MicroRNA-92a-3p overexpression in peripheral blood mononuclear cells is an independent predictor of prolonged overall survival of patients with chronic lymphocytic leukemia. Leuk. Lymphoma.

[12] Diamantopoulos M.A., Kontos C.K., Kerimis D., Papadopoulos I.N., Scorilas A. (2017). Upregulated miR-16 expression is an independent indicator of relapse and poor overall survival of colorectal adenocarcinoma patients. Clin. Chem. Lab. Med..

[13] Masoud V., Pagès G. (2017). Targeted therapies in breast cancer: New challenges to fight against resistance. World J. Clin. Oncol..

[14] Mollaei H., Safaralizadeh R., Rostami Z. (2019). MicroRNA replacement therapy in cancer. J. Cell. Physiol..

[15] Boti M.A., Diamantopoulos M.A., Scorilas A. (2025). RNA-Targeting Techniques: A Comparative Analysis of Modern Approaches for RNA Manipulation in Cancer Research and Therapeutics. Genes.

[16] Boti M.A., Athanasopoulou K., Adamopoulos P.G., Sideris D.C., Scorilas A. (2023). Recent Advances in Genome-Engineering Strategies. Genes.

[17] Kalinowski L., Saunus J.M., McCart Reed A.E., Lakhani S.R. (2019). Breast Cancer Heterogeneity in Primary and Metastatic Disease. Adv. Exp. Med. Biol..

[18] Zhao N., Rosen J.M. (2022). Breast cancer heterogeneity through the lens of single-cell analysis and spatial pathologies. Semin. Cancer Biol..

[19] Bray F., Laversanne M., Sung H., Ferlay J., Siegel R.L., Soerjomataram I., Jemal A. (2024). Global cancer statistics 2022: GLOBOCAN estimates of incidence and mortality worldwide for 36 cancers in 185 countries. CA Cancer J. Clin..

[20] Organisation W.H. Breast Cancer. https://www.who.int/news-room/fact-sheets/detail/breast-cancer.

[21] Kim J., Harper A., McCormack V., Sung H., Houssami N., Morgan E., Mutebi M., Garvey G., Soerjomataram I., Fidler-Benaoudia M.M. (2025). Global patterns and trends in breast cancer incidence and mortality across 185 countries. Nat. Med..

[22] Arnold M., Morgan E., Rumgay H., Mafra A., Singh D., Laversanne M., Vignat J., Gralow J.R., Cardoso F., Siesling S. (2022). Current and future burden of breast cancer: Global statistics for 2020 and 2040. Breast.

[23] Katsura C., Ogunmwonyi I., Kankam H.K., Saha S. (2022). Breast cancer: Presentation, investigation and management. Br. J. Hosp. Med..

[24] Koh J., Kim M.J. (2019). Introduction of a New Staging System of Breast Cancer for Radiologists: An Emphasis on the Prognostic Stage. Korean J. Radiol..

[25] Kudela E., Samec M., Koklesova L., Liskova A., Kubatka P., Kozubik E., Rokos T., Pribulova T., Gabonova E., Smolar M. (2020). miRNA Expression Profiles in Luminal A Breast Cancer-Implications in Biology, Prognosis, and Prediction of Response to Hormonal Treatment. Int. J. Mol. Sci..

[26] Xiong X., Zheng L.W., Ding Y., Chen Y.F., Cai Y.W., Wang L.P., Huang L., Liu C.C., Shao Z.M., Yu K.D. (2025). Breast cancer: Pathogenesis and treatments. Signal Transduct. Target. Ther..

[27] Dai X., Cheng H., Bai Z., Li J. (2017). Breast Cancer Cell Line Classification and Its Relevance with Breast Tumor Subtyping. J. Cancer.

[28] Yin L., Duan J.J., Bian X.W., Yu S.C. (2020). Triple-negative breast cancer molecular subtyping and treatment progress. Breast Cancer Res. BCR.

[29] Arun R.P., Cahill H.F., Marcato P. (2022). Breast Cancer Subtype-Specific miRNAs: Networks, Impacts, and the Potential for Intervention. Biomedicines.

[30] Tang J., Ahmad A., Sarkar F.H. (2012). The role of microRNAs in breast cancer migration, invasion and metastasis. Int. J. Mol. Sci..

[31] Ghafouri-Fard S., Khanbabapour Sasi A., Abak A., Shoorei H., Khoshkar A., Taheri M. (2021). Contribution of miRNAs in the Pathogenesis of Breast Cancer. Front. Oncol..

[32] Haakensen V.D., Nygaard V., Greger L., Aure M.R., Fromm B., Bukholm I.R., Lüders T., Chin S.F., Git A., Caldas C. (2016). Subtype-specific micro-RNA expression signatures in breast cancer progression. Int. J. Cancer.

[33] Liu P., Ye F., Xie X., Li X., Tang H., Li S., Huang X., Song C., Wei W., Xie X. (2016). mir-101-3p is a key regulator of tumor metabolism in triple negative breast cancer targeting AMPK. Oncotarget.

[34] Hong H.-C., Chuang C.-H., Huang W.-C., Weng S.-L., Chen C.-H., Chang K.-H., Liao K.-W., Huang H.-D. (2020). A panel of eight microRNAs is a good predictive parameter for triple-negative breast cancer relapse. Theranostics.

[35] Peng F., Zhang Y., Wang R., Zhou W., Zhao Z., Liang H., Qi L., Zhao W., Wang H., Wang C. (2016). Identification of differentially expressed miRNAs in individual breast cancer patient and application in personalized medicine. Oncogenesis.

[36] Søkilde R., Persson H., Ehinger A., Pirona A.C., Fernö M., Hegardt C., Larsson C., Loman N., Malmberg M., Rydén L. (2019). Refinement of breast cancer molecular classification by miRNA expression profiles. BMC Genom..

[37] Amorim M., Lobo J., Fontes-Sousa M., Estevão-Pereira H., Salta S., Lopes P., Coimbra N., Antunes L., Palma de Sousa S., Henrique R. (2019). Predictive and prognostic value of selected MicroRNAs in luminal breast cancer. Front. Genet..

[38] McDermott A.M., Miller N., Wall D., Martyn L.M., Ball G., Sweeney K.J., Kerin M.J. (2014). Identification and validation of oncologic miRNA biomarkers for luminal A-like breast cancer. PLoS ONE.

[39] Newie I., Søkilde R., Persson H., Grabau D., Rego N., Kvist A., von Stedingk K., Axelson H., Borg Å., Vallon-Christersson J. (2014). The HER2-encoded miR-4728-3p regulates ESR1 through a non-canonical internal seed interaction. PLoS ONE.

[40] Luo Y., Wang X., Niu W., Wang H., Wen Q., Fan S., Zhao R., Li Z., Xiong W., Peng S. (2017). Elevated microRNA-125b levels predict a worse prognosis in HER2-positive breast cancer patients. Oncol. Lett..

[41] Persson H., Kvist A., Rego N., Staaf J., Vallon-Christersson J., Luts L., Loman N., Jonsson G., Naya H., Hoglund M. (2011). Identification of new microRNAs in paired normal and tumor breast tissue suggests a dual role for the ERBB2/Her2 gene. Cancer Res..

[42] Tashkandi H., Shah N., Patel Y., Chen H. (2015). Identification of new miRNA biomarkers associated with HER2-positive breast cancers. Oncoscience.

[43] Floros K.V., Lochmann T.L., Hu B., Monterrubio C., Hughes M.T., Wells J.D., Morales C.B., Ghotra M.S., Costa C., Souers A.J. (2018). Coamplification of miR-4728 protects HER2-amplified breast cancers from targeted therapy. Proc. Natl. Acad. Sci. USA.

[44] Moi L., Braaten T., Al-Shibli K., Lund E., Busund L.R. (2019). Differential expression of the miR-17-92 cluster and miR-17 family in breast cancer according to tumor type; results from the Norwegian Women and Cancer (NOWAC) study. J. Transl. Med..

[45] Yang F., Zhang W., Shen Y., Guan X. (2015). Identification of dysregulated microRNAs in triple-negative breast cancer (Review). Int. J. Oncol..

[46] Correia de Sousa M., Gjorgjieva M., Dolicka D., Sobolewski C., Foti M. (2019). Deciphering miRNAs’ Action through miRNA Editing. Int. J. Mol. Sci..

[47] Lin S., Gregory R.I. (2015). MicroRNA biogenesis pathways in cancer. Nat. Rev. Cancer.

[48] Bartel D.P. (2018). Metazoan MicroRNAs. Cell.

[49] Inoue J., Inazawa J. (2021). Cancer-associated miRNAs and their therapeutic potential. J. Hum. Genet..

[50] Hanahan D. (2022). Hallmarks of Cancer: New Dimensions. Cancer Discov..

[51] Li Y., Wang Y.W., Chen X., Ma R.R., Guo X.Y., Liu H.T., Jiang S.J., Wei J.M., Gao P. (2020). MicroRNA-4472 Promotes Tumor Proliferation and Aggressiveness in Breast Cancer by Targeting RGMA and Inducing EMT. Clin. Breast Cancer.

[52] Huang J., Zhou Q., Chen C., Chen L., Xu X., Wang Y., An Z., Lin C., Han H. (2021). MicroRNA miR-92a-3p regulates breast cancer cell proliferation and metastasis via regulating B-cell translocation gene 2 (BTG2). Bioengineered.

[53] Xu R., Guo C.X., Qian J., Yin F.X., Zhang M.L. (2025). Exosomal miR-155-5p Modulates Breast Cancer Proliferation and Metastasis Via NF-κB Activation. J. Biochem. Mol. Toxicol..

[54] Assiri A.A., Mourad N., Shao M., Kiel P., Liu W., Skaar T.C., Overholser B.R. (2019). MicroRNA 362-3p Reduces hERG-related Current and Inhibits Breast Cancer Cells Proliferation. Cancer Genom. Proteom..

[55] Yang H., Wang Z., Hu S., Chen L., Li W., Yang Z. (2023). miRNA-874-3p inhibits the migration, invasion and proliferation of breast cancer cells by targeting VDAC1. Aging.

[56] Yang K., Li D., Jia W., Song Y., Sun N., Wang J., Li H., Yin C. (2022). MiR-379-5p inhibits the proliferation, migration, and invasion of breast cancer by targeting KIF4A. Thorac. Cancer.

[57] Mohamed S.H., Kamal M.M., Reda A.M., Mesbah N.M., Abo-Elmatty D.M., Abdel-Hamed A.R. (2025). MicroRNA-205-5p inhibits the growth and migration of breast cancer through targeting Wnt/β-catenin co-receptor LRP6 and interacting with lncRNAs. Mol. Cell. Biochem..

[58] Chi Y., Wang F., Zhang T., Xu H., Zhang Y., Shan Z., Wu S., Fan Q., Sun Y. (2019). miR-516a-3p inhibits breast cancer cell growth and EMT by blocking the Pygo2/Wnt signalling pathway. J. Cell. Mol. Med..

[59] Li L., Zhang Y., Yang K., Liu W., Zhou Z., Xu Y. (2024). miRNA-449c-5p regulates the JAK-STAT pathway in inhibiting cell proliferation and invasion in human breast cancer cells by targeting ERBB2. Cancer Rep..

[60] Wang Z., Hu S., Li X., Liu Z., Han D., Wang Y., Wei L., Zhang G., Wang X. (2021). MiR-16-5p suppresses breast cancer proliferation by targeting ANLN. BMC Cancer.

[61] Lu Y., Wang K., Peng Y., Chen M., Zhong L., Huang L., Cheng F.U., Sheng X., Yang X., Ouyang M. (2025). Hsa-miR-214-3p inhibits breast cancer cell growth and improves the tumor immune microenvironment by downregulating B7H3. Oncol. Res..

[62] Ma S., Wei H., Wang C., Han J., Chen X., Li Y. (2021). MiR-26b-5p inhibits cell proliferation and EMT by targeting MYCBP in triple-negative breast cancer. Cell. Mol. Biol. Lett..

[63] Arabkari V., Barua D., Hossain M.M., Webber M., Smith T., Gupta A., Gupta S. (2023). miRNA-378 Is Downregulated by XBP1 and Inhibits Growth and Migration of Luminal Breast Cancer Cells. Int. J. Mol. Sci..

[64] Morana O., Wood W., Gregory C.D. (2022). The Apoptosis Paradox in Cancer. Int. J. Mol. Sci..

[65] Hassan A., Aubel C. (2025). The PI3K/Akt/mTOR Signaling Pathway in Triple-Negative Breast Cancer: A Resistance Pathway and a Prime Target for Targeted Therapies. Cancers.

[66] Li L., Wei D., Zhang J., Deng R., Tang J., Su D. (2022). miR-641 Inhibited Cell Proliferation and Induced Apoptosis by Targeting NUCKS1/PI3K/AKT Signaling Pathway in Breast Cancer. Comput. Math. Methods Med..

[67] Zhang Y., Li S., Cui X., Wang Y. (2023). microRNA-944 inhibits breast cancer cell proliferation and promotes cell apoptosis by reducing SPP1 through inactivating the PI3K/Akt pathway. Apoptosis An. Int. J. Program. Cell Death.

[68] Yadollahi-Farsani M., Amini-Farsani Z., Moayedi F., Khazaei N., Yaghoobi H. (2023). MiR-548k suppresses apoptosis in breast cancer cells by affecting PTEN/PI3K/AKT signaling pathway. IUBMB Life.

[69] Syed R.U., Banu H., Alshammrani A., Alshammari M.D., G S.K., Kadimpati K.K., Khalifa A.A.S., Aboshouk N.A.M., Almarir A.M., Hussain A. (2024). MicroRNA-21 (miR-21) in breast cancer: From apoptosis dysregulation to therapeutic opportunities. Pathol. Res. Pract..

[70] Liu T., Ye P., Ye Y., Han B. (2021). MicroRNA-216b targets HK2 to potentiate autophagy and apoptosis of breast cancer cells via the mTOR signaling pathway. Int. J. Biol. Sci..

[71] Li L., Yuan L., Luo J., Gao J., Guo J., Xie X. (2013). MiR-34a inhibits proliferation and migration of breast cancer through down-regulation of Bcl-2 and SIRT1. Clin. Exp. Med..

[72] Zhang B., Liu X.X., He J.R., Zhou C.X., Guo M., He M., Li M.F., Chen G.Q., Zhao Q. (2011). Pathologically decreased miR-26a antagonizes apoptosis and facilitates carcinogenesis by targeting MTDH and EZH2 in breast cancer. Carcinogenesis.

[73] Zhou M., Liu Z., Zhao Y., Ding Y., Liu H., Xi Y., Xiong W., Li G., Lu J., Fodstad O. (2010). MicroRNA-125b confers the resistance of breast cancer cells to paclitaxel through suppression of pro-apoptotic Bcl-2 antagonist killer 1 (Bak1) expression. J. Biol. Chem..

[74] Zhang C., Zhang J., Zhang A., Wang Y., Han L., You Y., Pu P., Kang C. (2010). PUMA is a novel target of miR-221/222 in human epithelial cancers. Int. J. Oncol..

[75] Khosravi Shahi P., Soria Lovelle A., Pérez Manga G. (2009). Tumoral angiogenesis and breast cancer. Clin. Transl. Oncol..

[76] Zhong M., Li N., Qiu X., Ye Y., Chen H., Hua J., Yin P., Zhuang G. (2020). TIPE regulates VEGFR2 expression and promotes angiogenesis in colorectal cancer. Int. J. Biol. Sci..

[77] Liu Z.-L., Chen H.-H., Zheng L.-L., Sun L.-P., Shi L. (2023). Angiogenic signaling pathways and anti-angiogenic therapy for cancer. Signal Transduct. Target. Ther..

[78] Hussen B.M., Salihi A., Abdullah S.T., Rasul M.F., Hidayat H.J., Hajiesmaeili M., Ghafouri-Fard S. (2022). Signaling pathways modulated by miRNAs in breast cancer angiogenesis and new therapeutics. Pathol. Res. Pract..

[79] Cabello P., Torres-Ruiz S., Adam-Artigues A., Forés-Martos J., Martínez M.T., Hernando C., Zazo S., Madoz-Gúrpide J., Rovira A., Burgués O. (2023). miR-146a-5p Promotes Angiogenesis and Confers Trastuzumab Resistance in HER2+ Breast Cancer. Cancers.

[80] Hunter S., Nault B., Ugwuagbo K.C., Maiti S., Majumder M. (2019). Mir526b and Mir655 Promote Tumour Associated Angiogenesis and Lymphangiogenesis in Breast Cancer. Cancers.

[81] Danza K., De Summa S., Pinto R., Pilato B., Palumbo O., Merla G., Simone G., Tommasi S. (2015). MiR-578 and miR-573 as potential players in BRCA-related breast cancer angiogenesis. Oncotarget.

[82] Zuo Y., Qu C., Tian Y., Wen Y., Xia S., Ma M. (2021). The HIF-1/SNHG1/miR-199a-3p/TFAM axis explains tumor angiogenesis and metastasis under hypoxic conditions in breast cancer. BioFactors.

[83] Resendiz-Hernández M., García-Hernández A.P., Silva-Cázares M.B., Coronado-Uribe R., Hernández-de la Cruz O.N., Arriaga-Pizano L.A., Prieto-Chávez J.L., Salinas-Vera Y.M., Ibarra-Sierra E., Ortiz-Martínez C. (2024). MicroRNA-204 Regulates Angiogenesis and Vasculogenic Mimicry in CD44+/CD24- Breast Cancer Stem-like Cells. Non-Coding RNA.

[84] Lu Y., Qin T., Li J., Wang L., Zhang Q., Jiang Z., Mao J. (2017). MicroRNA-140-5p inhibits invasion and angiogenesis through targeting VEGF-A in breast cancer. Cancer Gene Ther..

[85] Alhasan L. (2019). MiR-126 Modulates Angiogenesis in Breast Cancer by Targeting VEGF-A -mRNA. Asian Pac. J. Cancer Prev. APJCP.

[86] Wang Y., Zhang X., Zou C., Kung H.F., Lin M.C., Dress A., Wardle F., Jiang B.H., Lai L. (2016). miR-195 inhibits tumor growth and angiogenesis through modulating IRS1 in breast cancer. Biomed. Pharmacother. Biomed. Pharmacother..

[87] Li X., Zou Z.Z., Wen M., Xie Y.Z., Peng K.J., Luo T., Liu S.Y., Gu Q., Li J.J., Luo Z.Y. (2020). ZLM-7 inhibits the occurrence and angiogenesis of breast cancer through miR-212-3p/Sp1/VEGFA signal axis. Mol. Med..

[88] Tao Q., Qi Y., Gu J., Yu D., Lu Y., Liu J., Liang X. (2022). Breast cancer cells-derived Von Willebrand Factor promotes VEGF-A-related angiogenesis through PI3K/Akt-miR-205-5p signaling pathway. Toxicol. Appl. Pharmacol..

[89] Novikov N.M., Zolotaryova S.Y., Gautreau A.M., Denisov E.V. (2021). Mutational drivers of cancer cell migration and invasion. Br. J. Cancer.

[90] Huang Z., Zhen S., Jin L., Chen J., Han Y., Lei W., Zhang F. (2023). miRNA-1260b Promotes Breast Cancer Cell Migration and Invasion by Downregulating CCDC134. Curr. Gene Ther..

[91] Li H., Li H.H., Chen Q., Wang Y.Y., Fan C.C., Duan Y.Y., Huang Y., Zhang H.M., Li J.P., Zhang X.Y. (2022). miR-142-5p Inhibits Cell Invasion and Migration by Targeting DNMT1 in Breast Cancer. Oncol. Res..

[92] Tam W.L., Weinberg R.A. (2013). The epigenetics of epithelial-mesenchymal plasticity in cancer. Nat. Med..

[93] Pan Y., Zhao Y., Lihui L., Xie Y., Zou Q. (2021). MiR-337-3p suppresses migration and invasion of breast cancer cells by downregulating ESRP1. Acta Histochem..

[94] Wang L., Guo Z., Zhang S., Zhang X., Sang M., Liu Y., Shan B. (2023). MIR-518a-5p Targets ZEB2 to Suppress the Migration and Invasion of Breast-cancer Cells. Altern. Ther. Health Med..

[95] Fardi M., Alivand M., Baradaran B., Farshdousti Hagh M., Solali S. (2019). The crucial role of ZEB2: From development to epithelial-to-mesenchymal transition and cancer complexity. J. Cell. Physiol..

[96] Zhao C., Ling X., Li X., Hou X., Zhao D. (2019). MicroRNA-138-5p inhibits cell migration, invasion and EMT in breast cancer by directly targeting RHBDD1. Breast Cancer.

[97] Li Y., Wang Y., Fan H., Zhang Z., Li N. (2018). miR-125b-5p inhibits breast cancer cell proliferation, migration and invasion by targeting KIAA1522. Biochem. Biophys. Res. Commun..

[98] Xie Z.H., Yu J., Shang L., Zhu Y.Q., Hao J.J., Cai Y., Xu X., Zhang Y., Wang M.R. (2017). KIA1522 overexpression promotes tumorigenicity and metastasis of esophageal cancer cells through potentiating the ERK activity. OncoTargets Ther..

[99] Zhang H., Wang M., Lang Z., Liu H., Liu J., Ma L. (2022). MiR-135a-5p suppresses breast cancer cell proliferation, migration, and invasion by regulating BAG3. Clinics.

[100] Jiang Q., He M., Ma M.T., Wu H.Z., Yu Z.J., Guan S., Jiang L.Y., Wang Y., Zheng D.D., Jin F. (2016). MicroRNA-148a inhibits breast cancer migration and invasion by directly targeting WNT-1. Oncol. Rep..

[101] Wm Nor W., Chung I., Said N. (2021). MicroRNA-548m Suppresses Cell Migration and Invasion by Targeting Aryl Hydrocarbon Receptor in Breast Cancer Cells. Oncol. Res..

[102] Han M., Wang Y., Guo G., Li L., Dou D., Ge X., Lv P., Wang F., Gu Y. (2018). microRNA-30d mediated breast cancer invasion, migration, and EMT by targeting KLF11 and activating STAT3 pathway. J. Cell. Biochem..

[103] Huang L., Tang X., Shi X., Su L. (2020). miR-532-5p promotes breast cancer proliferation and migration by targeting RERG. Exp. Ther. Med..

[104] Liang Z., Liu L., Gao R., Che C., Yang G. (2022). Downregulation of exosomal miR-7-5p promotes breast cancer migration and invasion by targeting RYK and participating in the atypical WNT signalling pathway. Cell. Mol. Biol. Lett..

[105] Wang Y., Jia J., Wang F., Fang Y., Yang Y., Zhou Q., Yuan W., Gu X., Hu J., Yang S. (2024). Pre-metastatic niche: Formation, characteristics and therapeutic implication. Signal Transduct. Target. Ther..

[106] Shen S., Song Y., Zhao B., Xu Y., Ren X., Zhou Y., Sun Q. (2021). Cancer-derived exosomal miR-7641 promotes breast cancer progression and metastasis. Cell Commun. Signal. CCS.

[107] Wang Y., Shi S., Wang Y., Zhang X., Liu X., Li J., Li P., Du L., Wang C. (2022). miR-223-3p targets FBXW7 to promote epithelial-mesenchymal transition and metastasis in breast cancer. Thorac. Cancer.

[108] Lu Y., Hu X., Yang X. (2021). miR-934 promotes breast cancer metastasis by regulation of PTEN and epithelial–mesenchymal transition. Tissue Cell.

[109] Lv Y., Li Y., Zhou J., Liu X., Wang D., Wang D., Tong D., Wang S., An H., Kang X. (2025). Exosomal miR-122-5p for regulation of secretory functions of fibroblasts and promotion of breast cancer metastasis by targeting MKP-2: An experimental study. Cancer Biol. Ther..

[110] Zeng F., Yao M., Wang Y., Zheng W., Liu S., Hou Z., Cheng X., Sun S., Li T., Zhao H. (2022). Fatty acid β-oxidation promotes breast cancer stemness and metastasis via the miRNA-328-3p-CPT1A pathway. Cancer Gene Ther..

[111] Akshaya R.L., Saranya I., Salomi G.M., Shanthi P., Ilangovan R., Venkataraman P., Selvamurugan N. (2024). In vivo validation of the functional role of MicroRNA-4638-3p in breast cancer bone metastasis. J. Cancer Res. Clin. Oncol..

[112] Choi S., An H.J., Yeo H.J., Sung M.J., Oh J., Lee K., Lee S.A., Kim S.K., Kim J., Kim I. (2024). MicroRNA-606 inhibits the growth and metastasis of triple-negative breast cancer by targeting Stanniocalcin 1. Oncol. Rep..

[113] Li S.-Y., Zhang N., Zhang H., Wang N., Du Y.-Y., Li H.-N., Huang C.-S., Li X.-R. (2024). Deciphering the TCF19/miR-199a-5p/SP1/LOXL2 pathway: Implications for breast cancer metastasis and epithelial-mesenchymal transition. Cancer Lett..

[114] Samaeekia R., Adorno-Cruz V., Bockhorn J., Chang Y.F., Huang S., Prat A., Ha N., Kibria G., Huo D., Zheng H. (2017). miR-206 Inhibits Stemness and Metastasis of Breast Cancer by Targeting MKL1/IL11 Pathway. Clin. Cancer Res. An. Off. J. Am. Assoc. Cancer Res..

[115] Thammaiah C.K., Jayaram S. (2016). Role of let-7 family microRNA in breast cancer. Non-Coding RNA Res..

[116] Manore S.G., Doheny D.L., Wong G.L., Lo H.W. (2022). IL-6/JAK/STAT3 Signaling in Breast Cancer Metastasis: Biology and Treatment. Front. Oncol..

[117] Gu P., Ding W., Zhu W., Shen L., Zhang L., Wang W., Wang R., Wang W., Wang Y., Yan B. (2024). MIR4435-2HG: A novel biomarker for triple-negative breast cancer diagnosis and prognosis, activating cancer-associated fibroblasts and driving tumor invasion through EMT associated with JNK/c-Jun and p38 MAPK signaling pathway activation. Int. Immunopharmacol..

[118] Xiang Y., Liao X.H., Yu C.X., Yao A., Qin H., Li J.P., Hu P., Li H., Guo W., Gu C.J. (2017). MiR-93-5p inhibits the EMT of breast cancer cells via targeting MKL-1 and STAT3. Exp. Cell Res..

[119] Wang N., Wei L., Huang Y., Wu Y., Su M., Pang X., Wang N., Ji F., Zhong C., Chen T. (2017). miR520c blocks EMT progression of human breast cancer cells by repressing STAT3. Oncol. Rep..

[120] van Schooneveld E., Wildiers H., Vergote I., Vermeulen P.B., Dirix L.Y., Van Laere S.J. (2015). Dysregulation of microRNAs in breast cancer and their potential role as prognostic and predictive biomarkers in patient management. Breast Cancer Res. BCR.

[121] Hosseinahli N., Aghapour M., Duijf P.H.G., Baradaran B. (2018). Treating cancer with microRNA replacement therapy: A literature review. J. Cell. Physiol..

[122] Wang Z., Clifton N.J. (2011). The guideline of the design and validation of MiRNA mimics. MicroRNA and Cancer.

[123] Nogimori T., Furutachi K., Ogami K., Hosoda N., Hoshino S.I. (2019). A novel method for stabilizing microRNA mimics. Biochem. Biophys. Res. Commun..

[124] Zhang M., Wang H., Zhang X., Liu F. (2021). miR-653-5p suppresses the growth and migration of breast cancer cells by targeting MAPK6. Mol. Med. Rep..

[125] Garzon R., Marcucci G., Croce C.M. (2010). Targeting microRNAs in cancer: Rationale, strategies and challenges. Nat. Rev. Drug Discov..

[126] Henry J.C., Azevedo-Pouly A.C., Schmittgen T.D. (2011). MicroRNA replacement therapy for cancer. Pharm. Res..

[127] Tavanafar F., Safaralizadeh R., Hosseinpour-Feizi M.A., Mansoori B., Shanehbandi D., Mohammadi A., Baradaran B. (2017). Restoration of miR-143 expression could inhibit migration and growth of MDA-MB-468 cells through down-regulating the expression of invasion-related factors. Biomed. Pharmacother. Biomed. Pharmacother..

[128] Yadav P., Sharma P., Sundaram S., Venkatraman G., Bera A.K., Karunagaran D. (2021). SLC7A11/ xCT is a target of miR-5096 and its restoration partially rescues miR-5096-mediated ferroptosis and anti-tumor effects in human breast cancer cells. Cancer Lett..

[129] Zhou J., Xiang A.Z., Guo J.F., Cui H.D. (2019). miR-30b suppresses the progression of breast cancer through inhibition of the PI3K/Akt signaling pathway by targeting Derlin-1. Transl. Cancer Res..

[130] Tavakolpour V., Safari-Kharkeshi M., Soleimanpour-Lichaei H.R., Gardaneh M., Kouhkan F. (2025). MicroRNA-34a enhances immune response and suppresses PI3K/AKT signaling through targeting PD-L1 in triple-negative breast cancer. Gene Rep..

[131] Ebert M.S., Sharp P.A. (2010). MicroRNA sponges: Progress and possibilities. RNA.

[132] Kluiver J., Slezak-Prochazka I., Smigielska-Czepiel K., Halsema N., Kroesen B.J., van den Berg A. (2012). Generation of MiRNA Sponge Constructs. Methods.

[133] Tay F.C., Lim J.K., Zhu H., Hin L.C., Wang S. (2015). Using artificial microRNA sponges to achieve microRNA loss-of-function in cancer cells. Adv. Drug Deliv. Rev..

[134] Panda A.C. (2018). Circular RNAs Act as miRNA Sponges. Adv. Exp. Med. Biol..

[135] Kulcheski F.R., Christoff A.P., Margis R. (2016). Circular RNAs are miRNA sponges and can be used as a new class of biomarker. J. Biotechnol..

[136] Liang A.L., Zhang T.T., Zhou N., Wu C.Y., Lin M.H., Liu Y.J. (2016). MiRNA-10b sponge: An anti-breast cancer study in vitro. Oncol. Rep..

[137] Gao S., Tian H., Guo Y., Li Y., Guo Z., Zhu X., Chen X. (2015). miRNA oligonucleotide and sponge for miRNA-21 inhibition mediated by PEI-PLL in breast cancer therapy. Acta Biomater..

[138] Rama A.R., Lara P., Mesas C., Quiñonero F., Vélez C., Melguizo C., Prados J. (2022). Circular Sponge against miR-21 Enhances the Antitumor Activity of Doxorubicin against Breast Cancer Cells. Int. J. Mol. Sci..

[139] Quirico L., Rizzolio S., Bertone S., Cirillo P.D.R., Savino A., Vitale N., Catuogno S., Esposito C.L., Stadler M.B., Defilippi P. (2025). The chimeric aptamer axl-miR-214sponge inhibits breast cancer and melanoma dissemination. Mol. Ther. J. Am. Soc. Gene Ther..

[140] Bravo-Estupiñan D.M., Geiß C., Arias-Arias J.L., Montaño-Samaniego M., Chinchilla-Monge R., Marín-Müller C., Quirós-Barrantes S., Régnier-Vigouroux A., Ibáñez-Hernández M., Mora-Rodríguez R.A. (2025). A Synthetic Sponge System Against miRNAs of the miR-17/92 Cluster Targets Transcriptional MYC Dosage Compensation in Aneuploid Cancer. Cells.

[141] Jung J., Yeom C., Choi Y.S., Kim S., Lee E., Park M.J., Kang S.W., Kim S.B., Chang S. (2015). Simultaneous inhibition of multiple oncogenic miRNAs by a multi-potent microRNA sponge. Oncotarget.

[142] Lennox K.A., Behlke M.A. (2010). A direct comparison of anti-microRNA oligonucleotide potency. Pharm. Res..

[143] Weiler J., Hunziker J., Hall J. (2006). Anti-miRNA oligonucleotides (AMOs): Ammunition to target miRNAs implicated in human disease?. Gene Ther..

[144] Davis S., Lollo B., Freier S., Esau C. (2006). Improved targeting of miRNA with antisense oligonucleotides. Nucleic Acids Res..

[145] Lima J.F., Cerqueira L., Figueiredo C., Oliveira C., Azevedo N.F. (2018). Anti-miRNA oligonucleotides: A comprehensive guide for design. RNA Biol..

[146] Pagoni M., Cava C., Sideris D.C., Avgeris M., Zoumpourlis V., Michalopoulos I., Drakoulis N. (2023). miRNA-Based Technologies in Cancer Therapy. J. Pers. Med..

[147] Gong Y., He T., Yang L., Yang G., Chen Y., Zhang X. (2015). The role of miR-100 in regulating apoptosis of breast cancer cells. Sci. Rep..

[148] Kamali M.J., Saeedi F., Khoshghiafeh A., Mir M.A., Aram C., Ahmadifard M. (2025). Therapeutic targeting of triple-negative breast cancer: A multi-model evaluation of LNA-anti-miR-19b-3p and small molecule inhibitors. Comput. Biol. Med..

[149] Naseri Z., Oskuee R.K., Forouzandeh-Moghadam M., Jaafari M.R. (2020). Delivery of LNA-antimiR-142-3p by Mesenchymal Stem Cells-Derived Exosomes to Breast Cancer Stem Cells Reduces Tumorigenicity. Stem Cell Rev. Rep..

[150] Khoder A.I., El-Sayed I.H., Ali Y.B.M. (2025). Targeting miR-32-5p suppresses c-MYC-driven proliferation and induces apoptosis in MCF-7 breast cancer cells. Med. Oncol..

[151] Okumura S., Hirano Y., Komatsu Y. (2021). Stable duplex-linked antisense targeting miR-148a inhibits breast cancer cell proliferation. Sci. Rep..

[152] Garneau J.E., Dupuis M.-È., Villion M., Romero D.A., Barrangou R., Boyaval P., Fremaux C., Horvath P., Magadán A.H., Moineau S. (2010). The CRISPR/Cas bacterial immune system cleaves bacteriophage and plasmid DNA. Nature.

[153] Bhaya D., Davison M., Barrangou R. (2011). CRISPR-Cas systems in bacteria and archaea: Versatile small RNAs for adaptive defense and regulation. Annu. Rev. Genet..

[154] Hussen B.M., Rasul M.F., Abdullah S.R., Hidayat H.J., Faraj G.S.H., Ali F.A., Salihi A., Baniahmad A., Ghafouri-Fard S., Rahman M. (2023). Targeting miRNA by CRISPR/Cas in cancer: Advantages and challenges. Mil. Med. Res..

[155] Mintz R.L., Gao M.A., Lo K., Lao Y.H., Li M., Leong K.W. (2018). CRISPR Technology for Breast Cancer: Diagnostics, Modeling, and Therapy. Adv. Biosyst..

[156] Karn V., Sandhya S., Hsu W., Parashar D., Singh H.N., Jha N.K., Gupta S., Dubey N.K., Kumar S. (2022). CRISPR/Cas9 system in breast cancer therapy: Advancement, limitations and future scope. Cancer Cell Int..

[157] Behrouzian Fard G., Ahmadi M.H., Gholamin M., Amirfakhrian R., Saberi Teimourian E., Karimi M.A., Hosseini Bafghi M. (2024). CRISPR-Cas9 technology: As an efficient genome modification tool in the cancer diagnosis and treatment. Biotechnol. Bioeng..

[158] Yang H., Patel D.J. (2024). Structures, mechanisms and applications of RNA-centric CRISPR-Cas13. Nat. Chem. Biol..

[159] Zhang H., Qin C., An C., Zheng X., Wen S., Chen W., Liu X., Lv Z., Yang P., Xu W. (2021). Application of the CRISPR/Cas9-based gene editing technique in basic research, diagnosis, and therapy of cancer. Mol. Cancer.

[160] Hannafon B.N., Cai A., Calloway C.L., Xu Y.F., Zhang R., Fung K.M., Ding W.Q. (2019). miR-23b and miR-27b are oncogenic microRNAs in breast cancer: Evidence from a CRISPR/Cas9 deletion study. BMC Cancer.

[161] Yi B., Wang S., Wang X., Liu Z., Zhang C., Li M., Gao S., Wei S., Bae S., Stringer-Reasor E. (2022). CRISPR interference and activation of the microRNA-3662-HBP1 axis control progression of triple-negative breast cancer. Oncogene.

[162] Uddin F., Rudin C.M., Sen T. (2020). CRISPR Gene Therapy: Applications, Limitations, and Implications for the Future. Front. Oncol..

[163] Heidersbach A.J., Dorighi K.M., Gomez J.A., Jacobi A.M., Haley B. (2023). A versatile, high-efficiency platform for CRISPR-based gene activation. Nat. Commun..

[164] Bendixen L., Jensen T.I., Bak R.O. (2023). CRISPR-Cas-mediated transcriptional modulation: The therapeutic promises of CRISPRa and CRISPRi. Mol. Ther. J. Am. Soc. Gene Ther..

[165] Nishimasu H., Ran F.A., Hsu P.D., Konermann S., Shehata S.I., Dohmae N., Ishitani R., Zhang F., Nureki O. (2014). Crystal Structure of Cas9 in Complex with Guide RNA and Target DNA. Cell.

[166] Cai R., Lv R., Shi X., Yang G., Jin J. (2023). CRISPR/dCas9 Tools: Epigenetic Mechanism and Application in Gene Transcriptional Regulation. Int. J. Mol. Sci..

[167] Rahman M.M., Tollefsbol T.O. (2021). Targeting cancer epigenetics with CRISPR-dCAS9: Principles and prospects. Methods.

[168] Kang T., Bleris L. (2026). Cellular-state control using ribozyme-scaffolded miRNA-sensing and CRISPR-mediated actuation. Cell Rep. Methods.

[169] Shu W.-J., Ma Z., Jia L., Guo B., Tian X., He C., Wang F. (2025). MiR-ON-CRISPR: A microRNA-activated CRISPR–dCas9 system for precise gene therapy in living cells and mouse models of sepsis. Nucleic Acids Res..

[170] Rajanathadurai J., Perumal E., Sindya J. (2024). Advances in targeting cancer epigenetics using CRISPR-dCas9 technology: A comprehensive review and future prospects. Funct. Integr. Genom..

[171] Zahraei M., Azimi Y., Karimipour M., Rahimi-Jamnani F., Valizadeh V., Azizi M. (2025). CRISPR/dCas9-TET1–mediated epigenetic editing reactivates miR-200c in breast cancer cells. Sci. Rep..

[172] Yang Y., Xu J., Ge S., Lai L. (2021). CRISPR/Cas: Advances, Limitations, and Applications for Precision Cancer Research. Front. Med..

[173] Guo C., Ma X., Gao F., Guo Y. (2023). Off-target effects in CRISPR/Cas9 gene editing. Front. Bioeng. Biotechnol..

[174] Raghavan R., Friedrich M.J., King I., Vo S.C.-D., Strebinger D., Lash B., Kilian M., Platten M., Macrae R.K., Song Y. (2025). Rational engineering of minimally immunogenic nucleases for gene therapy. Nat. Commun..

[175] Wagner D.L., Amini L., Wendering D.J., Burkhardt L.-M., Akyüz L., Reinke P., Volk H.-D., Schmueck-Henneresse M. (2019). High prevalence of *Streptococcus pyogenes* Cas9-reactive T cells within the adult human population. Nat. Med..

[176] Ewaisha R., Anderson K.S. (2023). Immunogenicity of CRISPR therapeutics-Critical considerations for clinical translation. Front. Bioeng. Biotechnol..

[177] Chang T.C., Wentzel E.A., Kent O.A., Ramachandran K., Mullendore M., Lee K.H., Feldmann G., Yamakuchi M., Ferlito M., Lowenstein C.J. (2007). Transactivation of miR-34a by p53 broadly influences gene expression and promotes apoptosis. Mol. Cell.

[178] Fujita Y., Kojima K., Hamada N., Ohhashi R., Akao Y., Nozawa Y., Deguchi T., Ito M. (2008). Effects of miR-34a on cell growth and chemoresistance in prostate cancer PC3 cells. Biochem. Biophys. Res. Commun..

[179] Raver-Shapira N., Marciano E., Meiri E., Spector Y., Rosenfeld N., Moskovits N., Bentwich Z., Oren M. (2007). Transcriptional activation of miR-34a contributes to p53-mediated apoptosis. Mol. Cell.

[180] Li X.J., Ji M.H., Zhong S.L., Zha Q.B., Xu J.J., Zhao J.H., Tang J.H. (2012). MicroRNA-34a modulates chemosensitivity of breast cancer cells to adriamycin by targeting Notch1. Arch. Med. Res..

[181] Shin J., Xie D., Zhong X.P. (2013). MicroRNA-34a enhances T cell activation by targeting diacylglycerol kinase ζ. PLoS ONE.

[182] Wang X., Li J., Dong K., Lin F., Long M., Ouyang Y., Wei J., Chen X., Weng Y., He T. (2015). Tumor suppressor miR-34a targets PD-L1 and functions as a potential immunotherapeutic target in acute myeloid leukemia. Cell. Signal..

[183] Kouhestani S.D., Khalili S., Razi A., Aghili M., Moghadam M.F. (2025). Ectopic expression of miR-34a/-328 sensitizes breast cancer stem cells to gamma rays/doxorubicin by BCL2/ABCG2 targeting. Mol. Biol. Rep..

[184] Hong D.S., Kang Y.-K., Borad M., Sachdev J., Ejadi S., Lim H.Y., Brenner A.J., Park K., Lee J.-L., Kim T.-Y. (2020). Phase 1 study of MRX34, a liposomal miR-34a mimic, in patients with advanced solid tumours. Br. J. Cancer.

[185] Wu Z., Cai X., Huang C., Xu J., Liu A. (2016). miR-497 suppresses angiogenesis in breast carcinoma by targeting HIF-1α. Oncol. Rep..

[186] Zhu Z., Wang S., Zhu J., Yang Q., Dong H., Huang J. (2016). MicroRNA-544 down-regulates both Bcl6 and Stat3 to inhibit tumor growth of human triple negative breast cancer. Biol. Chem..

[187] Bayraktar R., Pichler M., Kanlikilicer P., Ivan C., Bayraktar E., Kahraman N., Aslan B., Oguztuzun S., Ulasli M., Arslan A. (2017). MicroRNA 603 acts as a tumor suppressor and inhibits triple-negative breast cancer tumorigenesis by targeting elongation factor 2 kinase. Oncotarget.

[188] Yan Y., Li X.Q., Duan J.L., Bao C.J., Cui Y.N., Su Z.B., Xu J.R., Luo Q., Chen M., Xie Y. (2019). Nanosized functional miRNA liposomes and application in the treatment of TNBC by silencing Slug gene. Int. J. Nanomed..

[189] Okumura S., Hirano Y., Komatsu Y. (2020). Inhibition of breast cancer cell proliferation with anti-microRNA oligonucleotides flanked by interstrand cross-linked duplexes. Nucleosides Nucleotides Nucleic Acids.

[190] Shang M., Wu Y., Wang Y., Cai Y., Jin J., Yang Z. (2022). Dual antisense oligonucleotide targeting miR-21/miR-155 synergize photodynamic therapy to treat triple-negative breast cancer and inhibit metastasis. Biomed. Pharmacother..

[191] Gong L.G., Shi J.C., Shang J., Hao J.G., Du X. (2019). Effect of miR-34a on resistance to sunitinib in breast cancer by regulating the Wnt/β-catenin signaling pathway. Eur. Rev. Med. Pharmacol. Sci..

[192] Yu Y., Yin W., Yu Z.-H., Zhou Y.-J., Chi J.-R., Ge J., Cao X.-C. (2019). miR-190 enhances endocrine therapy sensitivity by regulating SOX9 expression in breast cancer. J. Exp. Clin. Cancer Res..

[193] Bustos M.A., Yokoe T., Shoji Y., Kobayashi Y., Mizuno S., Murakami T., Zhang X., Sekhar S.C., Kim S., Ryu S. (2023). MiR-181a targets STING to drive PARP inhibitor resistance in BRCA- mutated triple-negative breast cancer and ovarian cancer. Cell Biosci..

[194] Soung Y.H., Ju J., Chung J. (2023). The Sensitization of Triple-Negative Breast Cancers to Poly ADP Ribose Polymerase Inhibition Independent of BRCA1/2 Mutation Status by Chemically Modified microRNA-489. Cells.

[195] Tormo E., Adam-Artigues A., Ballester S., Pineda B., Zazo S., González-Alonso P., Albanell J., Rovira A., Rojo F., Lluch A. (2017). The role of miR-26a and miR-30b in HER2+ breast cancer trastuzumab resistance and regulation of the CCNE2 gene. Sci. Rep..

[196] Noyan S., Gurdal H., Gur Dedeoglu B. (2019). Involvement of miR-770-5p in trastuzumab response in HER2 positive breast cancer cells. PLoS ONE.

[197] Fu R., Tong J.S. (2020). miR-126 reduces trastuzumab resistance by targeting PIK3R2 and regulating AKT/mTOR pathway in breast cancer cells. J. Cell. Mol. Med..

[198] Wang Z., Peng Y., Zhou H., Zhang M., Ju D., Chen Z. (2025). MiRNA-based drugs: Challenges and delivery strategies. Appl. Microbiol. Biotechnol..

[199] Xue J., Chi Y., Chen Y., Huang S., Ye X., Niu J., Wang W., Pfeffer L.M., Shao Z.M., Wu Z.H. (2016). MiRNA-621 sensitizes breast cancer to chemotherapy by suppressing FBXO11 and enhancing p53 activity. Oncogene.

[200] Ahmadzada T., Reid G., McKenzie D.R. (2018). Fundamentals of siRNA and miRNA therapeutics and a review of targeted nanoparticle delivery systems in breast cancer. Biophys. Rev..

[201] Moraes F.C., Pichon C., Letourneur D., Chaubet F. (2021). miRNA Delivery by Nanosystems: State of the Art and Perspectives. Pharmaceutics.

[202] Yang N. (2015). An overview of viral and nonviral delivery systems for microRNA. Int. J. Pharm. Investig..

[203] Milone M.C., O’Doherty U. (2018). Clinical use of lentiviral vectors. Leukemia.

[204] Park T.S., Abate-Daga D., Zhang L., Zheng Z., Morgan R.A. (2014). Gamma-Retroviral Vector Design for the Co-Expression of Artificial microRNAs and Therapeutic Proteins. Nucleic Acid. Ther..

[205] Baum C., Düllmann J., Li Z., Fehse B., Meyer J., Williams D.A., von Kalle C. (2003). Side effects of retroviral gene transfer into hematopoietic stem cells. Blood.

[206] Laufs S., Guenechea G., Gonzalez-Murillo A., Zsuzsanna Nagy K., Luz Lozano M., del Val C., Jonnakuty S., Hotz-Wagenblatt A., Jens Zeller W., Bueren J.A. (2006). Lentiviral vector integration sites in human NOD/SCID repopulating cells. J. Gene Med..

[207] Crystal R.G. (2014). Adenovirus: The First Effective In Vivo Gene Delivery Vector. Hum. Gene Ther..

[208] Ricobaraza A., Gonzalez-Aparicio M., Mora-Jimenez L., Lumbreras S., Hernandez-Alcoceba R. (2020). High-capacity adenoviral vectors: Expanding the scope of gene therapy. Int. J. Mol. Sci..

[209] Watanabe M., Nishikawaji Y., Kawakami H., Kosai K.-I. (2021). Adenovirus biology, recombinant adenovirus, and adenovirus usage in gene therapy. Viruses.

[210] Syyam A., Nawaz A., Ijaz A., Sajjad U., Fazil A., Irfan S., Muzaffar A., Shahid M., Idrees M., Malik K. (2022). Adenovirus vector system: Construction, history and therapeutic applications. Biotechniques.

[211] Leikas A.J., Ylä-Herttuala S., Hartikainen J.E.K. (2023). Adenoviral Gene Therapy Vectors in Clinical Use—Basic Aspects with a Special Reference to Replication-Competent Adenovirus Formation and Its Impact on Clinical Safety. Int. J. Mol. Sci..

[212] Khan N., Cheemadan S., Saxena H., Bammidi S., Jayandharan G.R. (2020). MicroRNA-based recombinant AAV vector assembly improves efficiency of suicide gene transfer in a murine model of lymphoma. Cancer Med..

[213] Xie J., Burt D.R., Gao G. (2015). Adeno-associated virus-mediated microRNA delivery and therapeutics. Semin. Liver Dis..

[214] Mietzsch M., Hsi J., Nelson A.R., Khandekar N., Huang A.-M., Smith N.J.C., Zachary J., Potts L., Farrar M.A., Chipman P. (2025). Structural characterization of antibody-responses following Zolgensma treatment for AAV capsid engineering to expand patient cohorts. Nat. Commun..

[215] Pham Q., Glicksman J., Chatterjee A. (2024). Chemical approaches to probe and engineer AAV vectors. Nanoscale.

[216] Le Hars M., Joussain C., Jégu T., Epstein A.L. (2025). Non-replicative herpes simplex virus genomic and amplicon vectors for gene therapy—An update. Gene Ther..

[217] Ryan D.A., Federoff H.J. (2009). Immune responses to herpesviral vectors. Hum. Gene Ther..

[218] Park E.Y., Chang E., Lee E.J., Lee H.W., Kang H.G., Chun K.H., Woo Y.M., Kong H.K., Ko J.Y., Suzuki H. (2014). Targeting of miR34a-NOTCH1 axis reduced breast cancer stemness and chemoresistance. Cancer Res..

[219] Li Z., Min L., Chen L., Hu Y., Luan W., Li C., Xiong Q., Huang K. (2022). Therapeutic potential of anti-miR29a in breast cancer patients with type 2 diabetes: An in vitro and xenograft mouse-model study. Transl. Cancer Res..

[220] Deng L., Shang L., Bai S., Chen J., He X., Martin-Trevino R., Chen S., Li X.Y., Meng X., Yu B. (2014). MicroRNA100 inhibits self-renewal of breast cancer stem-like cells and breast tumor development. Cancer Res..

[221] Long X., Shi Y., Ye P., Guo J., Zhou Q., Tang Y. (2019). MicroRNA-99a Suppresses Breast Cancer Progression by Targeting FGFR3. Front. Oncol..

[222] Yan Y., Zhang F., Fan Q., Li X., Zhou K. (2014). Breast cancer-specific TRAIL expression mediated by miRNA response elements of let-7 and miR-122. Neoplasma.

[223] Trepel M., Körbelin J., Spies E., Heckmann M.B., Hunger A., Fehse B., Katus H.A., Kleinschmidt J.A., Müller O.J., Michelfelder S. (2015). Treatment of multifocal breast cancer by systemic delivery of dual-targeted adeno-associated viral vectors. Gene Ther..

[224] Huang K.C., Lai C.Y., Hung W.Z., Chang H.Y., Lin P.C., Chiang S.F., Ke T.W., Liang J.A., Shiau A.C., Yang P.C. (2023). A Novel Engineered AAV-Based Neoantigen Vaccine in Combination with Radiotherapy Eradicates Tumors. Cancer Immunol. Res..

[225] Wang C., Pan C., Yong H., Wang F., Bo T., Zhao Y., Ma B., He W., Li M. (2023). Emerging non-viral vectors for gene delivery. J. Nanobiotechnol..

[226] Ren S., Wang M., Wang C., Wang Y., Sun C., Zeng Z., Cui H., Zhao X. (2021). Application of Non-Viral Vectors in Drug Delivery and Gene Therapy. Polymers.

[227] Bai Z., Wei J., Yu C., Han X., Qin X., Zhang C., Liao W., Li L., Huang W. (2019). Non-viral nanocarriers for intracellular delivery of microRNA therapeutics. J. Mater. Chem. B.

[228] Lee S.W.L., Paoletti C., Campisi M., Osaki T., Adriani G., Kamm R.D., Mattu C., Chiono V. (2019). MicroRNA delivery through nanoparticles. J. Control. Release Off. J. Control. Release Soc..

[229] Dinger M.E., Mercer T.R., Mattick J.S. (2008). RNAs as extracellular signaling molecules. J. Mol. Endocrinol..

[230] Basu S.C., Basu M. (2008). Liposome Methods and Protocols.

[231] Duan Y., Dhar A., Patel C., Khimani M., Neogi S., Sharma P., Siva Kumar N., Vekariya R.L. (2020). A brief review on solid lipid nanoparticles: Part and parcel of contemporary drug delivery systems. RSC Adv..

[232] Shah R., Eldridge D., Palombo E., Harding I. (2015). Lipid Nanoparticles: Production, Characterization and Stability.

[233] Nsairat H., Khater D., Sayed U., Odeh F., Al Bawab A., Alshaer W. (2022). Liposomes: Structure, composition, types, and clinical applications. Heliyon.

[234] Yun Y., An J., Kim H.J., Choi H.K., Cho H.Y. (2025). Recent advances in functional lipid-based nanomedicines as drug carriers for organ-specific delivery. Nanoscale.

[235] Gareev I., Beylerli O., Tamrazov R., Ilyasova T., Shumadalova A., Du W., Yang B. (2023). Methods of miRNA delivery and possibilities of their application in neuro-oncology. Non-Coding RNA Res..

[236] Xue L., Tan Q., Xu J., Feng L., Li W., Yan L., Li Y. (2024). MiR-6838-5p overexpression inhibits proliferation of breast cancer MCF-7 cells by downregulating DDR1 expression. J. South. Med. Univ..

[237] Zhang H., Yu N., Chen Y., Yan K., Wang X. (2019). Cationic liposome codelivering PI3K pathway regulator improves the response of BRCA1-deficient breast cancer cells to PARP1 inhibition. J. Cell. Biochem..

[238] Chaudhari R., Patel V., Malvi B., Misra S.K., Kumar A. (2025). Maximising efficacy in HER2-positive breast cancer: Immunoliposomal co-delivery of miR155 inhibitor and paclitaxel for targeted therapy. J. Mater. Chem. B.

[239] Gorur A., Bayraktar R., Ivan C., Mokhlis H.A., Bayraktar E., Kahraman N., Karakas D., Karamil S., Kabil N.N., Kanlikilicer P. (2021). NcRNA therapy with miRNA-22-3p suppresses the growth of triple-negative breast cancer. Mol. Ther. Nucleic Acids.

[240] Ban E.-J., Kwon T.-H., Kim A. (2019). Delivery of therapeutic miRNA using polymer-based formulation. Drug Deliv. Transl. Res..

[241] Cheng C.J., Saltzman W.M. (2012). Polymer nanoparticle-mediated delivery of microRNA inhibition and alternative splicing. Mol. Pharm..

[242] Ahir M., Upadhyay P., Ghosh A., Sarker S., Bhattacharya S., Gupta P., Ghosh S., Chattopadhyay S., Adhikary A. (2020). Delivery of dual miRNA through CD44-targeted mesoporous silica nanoparticles for enhanced and effective triple-negative breast cancer therapy. Biomater. Sci..

[243] Bahreyni A., Alibolandi M., Ramezani M., Sarafan Sadeghi A., Abnous K., Taghdisi S.M. (2019). A novel MUC1 aptamer-modified PLGA-epirubicin-PβAE-antimir-21 nanocomplex platform for targeted co-delivery of anticancer agents in vitro and in vivo. Colloids Surf. B Biointerfaces.

[244] Devulapally R., Sekar N.M., Sekar T.V., Foygel K., Massoud T.F., Willmann J.K., Paulmurugan R. (2015). Polymer nanoparticles mediated codelivery of antimiR-10b and antimiR-21 for achieving triple negative breast cancer therapy. ACS Nano.

[245] Han T.Y., Huan M.L., Cai Z., He W., Zhou S.Y., Zhang B.L. (2023). Polymer-Initiating Caveolae-Mediated Endocytosis and GSH-Responsive MiR-34a Gene Delivery System for Enhanced Orthotopic Triple Negative Breast Cancer Therapy. Adv. Healthc. Mater..

[246] Zeng Y., Zhou Z., Fan M., Gong T., Zhang Z., Sun X. (2017). PEGylated Cationic Vectors Containing a Protease-Sensitive Peptide as a miRNA Delivery System for Treating Breast Cancer. Mol. Pharm..

[247] Yang X., Gao F., Zhang W., Li H., Huang X., Wei J., Bian J., Yang Y., Qian C., Sun M. (2020). “Star” miR-34a and CXCR4 antagonist based nanoplex for binary cooperative migration treatment against metastatic breast cancer. J. Control. Release.

[248] Zhang S., Pang S., Pei W., Zhu H., Shi Y., Liu Z., Mao L., Shi X., Tao S., Geng C. (2023). Layered Double Hydroxide-Loaded miR-30a for the Treatment of Breast Cancer In Vitro and In Vivo. ACS Omega.

[249] Shinge S.A.U., Xiao Y., Xia J., Liang Y., Duan L. (2022). New insights of engineering plant exosome-like nanovesicles as a nanoplatform for therapeutics and drug delivery. Extracell. Vesicles Circ. Nucleic Acids.

[250] Zuo Y., Zhang J., Wang X., Sun B., Tian S., Miao M. (2026). Plant-derived exosome-like nanovesicles: Dual-function platforms for anticancer therapy and drug delivery. J. Transl. Med..

[251] Kantarcioglu M. (2021). New therapeutic players on the horizon: Edible plant derived exosomes. Hepatol. Forum.

[252] Jin Z., Na J., Lin X., Jiao R., Liu X., Huang Y. (2024). Plant-derived exosome-like nanovesicles: A novel nanotool for disease therapy. Heliyon.

[253] Yi Q., Xu Z., Thakur A., Zhang K., Liang Q., Liu Y., Yan Y. (2023). Current understanding of plant-derived exosome-like nanoparticles in regulating the inflammatory response and immune system microenvironment. Pharmacol. Res..

[254] Yan G., Xiao Q., Zhao J., Chen H., Xu Y., Tan M., Peng L. (2024). Brucea javanica derived exosome-like nanovesicles deliver miRNAs for cancer therapy. J. Control. Release Off. J. Control. Release Soc..

[255] Shkryl Y., Tsydeneshieva Z., Menchinskaya E., Rusapetova T., Grishchenko O., Mironova A., Bulgakov D., Gorpenchenko T., Kazarin V., Tchernoded G. (2024). Exosome-like Nanoparticles, High in Trans-δ-Viniferin Derivatives, Produced from Grape Cell Cultures: Preparation, Characterization, and Anticancer Properties. Biomedicines.

[256] Amaldoss M.J.N., Yang J.L., Koshy P., Unnikrishnan A., Sorrell C.C. (2022). Inorganic nanoparticle-based advanced cancer therapies: Promising combination strategies. Drug Discov. Today.

[257] Tonga G.Y., Moyano D.F., Kim C.S., Rotello V.M. (2014). Inorganic nanoparticles for therapeutic delivery: Trials, tribulations and promise. Curr. Opin. Colloid Interface Sci..

[258] Chen L., Wang H., Zhang H., Mu W., Shi X., Chen X. (2025). Engineered gold nanoparticle-based miRNA precision regulation for tumor diagnosis and synergistic therapy. Smart Mater. Med..

[259] Crew E., Rahman S., Razzak-Jaffar A., Mott D., Kamundi M., Yu G., Tchah N., Lee J., Bellavia M., Zhong C.-J. (2012). MicroRNA Conjugated Gold Nanoparticles and Cell Transfection. Anal. Chem..

[260] Chaudhari R., Nasra S., Meghani N., Kumar A. (2022). MiR-206 conjugated gold nanoparticle based targeted therapy in breast cancer cells. Sci. Rep..

[261] Ramchandani D., Lee S.K., Yomtoubian S., Han M.S., Tung C.H., Mittal V. (2019). Nanoparticle Delivery of miR-708 Mimetic Impairs Breast Cancer Metastasis. Mol. Cancer Ther..

[262] Ren Y., Wang R., Gao L., Li K., Zhou X., Guo H., Liu C., Han D., Tian J., Ye Q. (2016). Sequential co-delivery of miR-21 inhibitor followed by burst release doxorubicin using NIR-responsive hollow gold nanoparticle to enhance anticancer efficacy. J. Control. Release.

